# Kidney and liver histology in tumour-induced rats exposed to non-contact electric fields

**DOI:** 10.12688/f1000research.110080.1

**Published:** 2023-02-01

**Authors:** Firman Alamsyah, Nisrina Firdausi, Subekti Evi Dwi Nugraheni, Ahmad Ghitha Fadhlurrahman, Luthfi Nurhidayat, Rarastoeti Pratiwi, Warsito Purwo Taruno

**Affiliations:** 1 Center for Medical Physics and Cancer Research, Ctech Labs Edwar Technology, Tangerang, Banten, 15143, Indonesia; 2 Faculty of Science and Technology, Universitas Al-Azhar Indonesia, Jl. Sisingamangaraja, Jakarta, 12110, Indonesia; 3 Research Center for Pharmaceutical Ingridients and Traditional Medicine, National Research and Innovation Agency Republic of Indonesia, Jl. Raya Jakarta-Bogor Km. 46, Cibinong West Java, 16911, Indonesia; 4 Medical Laboratory, Universitas Pendidikan Indonesia, Jl. Dr. Setiabudhi No. 229, Bandung West Java, 40154, Indonesia; 5 Faculty of Biology, Universitas Gadjah Mada, Sleman, DI Yogyakarta, 55281, Indonesia

**Keywords:** damages, histology, kidney, liver, non-contact electric field, ECCT

## Abstract

**Background:** There were an estimated 2.1 million breast cancer diagnoses in 2018 worldwide, which is about 11.6% of the total cancer incidence. A novel modality of cancer treatment based on exposure to non-contact electric fields has been developed to reduce cancer incidence. However, the safety of the electric field exposure was not fully investigated. Therefore, the purpose of this study is to observe the safety of the electric field exposure on renal and liver structure.

**Methods:** Female Sprague-Dawley rats were divided into one control group and three treatment groups. Animals were treated with 7,12-dimethylbenz[a]anthracene for mammary tumour induction and exposed to non-contact electric fields individually for 10 hours a day for three weeks. Fresh samples of the kidney and liver were collected for observing structural damage in both organs. The two organs were prepared for histopathological cross-sectioning using the paraffin method and Hematoxylin & Eosin staining followed by histological scoring using the post-examination masking method.

**Results:** The damages found in the kidney were the following: thickening of Bowman capsule, karyolysis, karyorrexhis, pyknosis, cloudy swelling, epithelial sloughing, inflammation, haemorrhage, and congestion. The number of inflammation and haemorrhage in the kidney structure of the placebo group was the lowest and significantly different from the three other groups. All damages in the kidney were also found in the liver, but each showed different levels of damage. The damages in the kidney and liver caused by the exposure were not significant.

**Conclusions:** The non-contact electric fields were not harmful to renal and liver structure in tumour-induced rats. Instead, it may increase the renal function in normal rats.

## Introduction

The knowledge that electric fields can induce biological effects was revealed in the 19 ^th^ century. Many studies were conducted which provided evidence that exposure to electric fields can generate alterations within living things. ^1^ Some studies have examined the effects generated by electric fields on cell functions. ^2^ Kirson *et al.* ^3^ also reported that electric field intensity within a cell is less than 10 V/cm, but in a cell membrane, it may gain 10 ^5^ V/cm. At the organ level, the kidney and liver have the dielectric property that exhibits a time-temperature dependence. ^4^^–^^6^ Therefore, they possess electrical conductivity and permittivity. ^5^^,^^6^

Porter *et al.* ^7^ explained that the knowledge of dielectric properties of biological tissues is valuable and useful in several applications of medical device, including cancer detection and treatment. As proof of that, the proliferation of cancer cells was successfully inhibited under exposure to intermediate frequency and low-intensity electric fields. ^3^^,^^8^^–^^11^ They use intermediate frequency to treat cancer because it specifically targets cancer cells and does not affect normal cells due to their higher membrane potential than that of cancer cells. ^12^^,^^13^ In our preliminary study, mammary tumour-induced mice that were exposed to intermediate frequency (100 kHz) and low-intensity (18 Volt peak to peak/Vpp) non-contact electric fields showed no histological alterations in mammary and skin tissues. ^8^ Furthermore, we developed non-contact electric fields to avoid dermatitis due to the direct contact between the electrodes and the skin, as reported by Kirson *et al.* ^3^ This novel modality has the potential to decrease the global cancer burden; 2.1 million people around the world were diagnosed with breast cancer in 2018, which is 11.6% of the total cancer incidence. ^14^

Although non-contact electric fields-based therapy has the potential to treat cancer, the safety of this kind of therapy when treating healthy tissues should be investigated. This is because injuries may occur after exposure to electric fields due to the dielectric property of the kidney and liver, which may interact with electrostatic waves. Therefore, it is important to investigate the abnormalities in the kidney and liver under exposure to electric fields during cancer treatment. The aim of this work was to investigate the safety of non-contact electric fields of strength 100 kHz-18 Vpp on the kidney and liver in the animal tumour model, with a focus on possible histological alterations in the organs. We hypothesised that exposure to non-contact electric fields would not affect the structure of the kidney and liver significantly. According to our knowledge, this is the first study investigating the abnormalities in the kidney and liver under exposure to 100 kHz intermediate frequency and low-intensity non-contact electric fields.

## Methods

### Experimental design

The experimental design and procedures, experimental animals, animal care and monitoring, housing and husbandry, sample size, inclusion and exclusion criteria, randomisation and blinding in this study were the same as the ones that have been previously reported. ^9^ For this study, 40 5-week-old healthy female Sprague Dawley (SD) rats ( *Rattus norvegicus,* Berkenhout 1769) weighing 50−80 g were used. This rat strain is one of the animals used as animal tumor models to study human breast cancer, since it has 98% genetic homology with humans. ^15^ These rats were provided by the Integrated Research and Testing Laboratory (LPPT) of Universitas Gadjah Mada (UGM), and never used for other studies. Rats that were sick or showing symptoms of disorder were excluded from the study. The rats were placed into polypropylene cages for one week of acclimatization. The cages were communal home cages with a size 50 × 40 cm ^2^ and the base was covered with rice hulls bedding. We prepared eight communal cages with each cage consisted of 5 animals. The lighting conditions in the animal’s room during the day came from light from the lamp, while at night it was total darkness (12L:12D photoperiod). We maintained room temperature to avoid dehydration during exposure to the electric field at 23–26°C with an average relative humidity of 81.09%.

We divided the animals into one control group (non-induction and non-therapy or NINT) and three treatment groups, namely placebo (non-induction and therapy or NIT), DMBA-induced mammary tumours without therapy (induction and non-therapy or INT), and DMBA induced mammary tumours with therapy (induction and therapy or IT) group. Using the Federer formula, the sample size in each group was calculated, in which 6 biological replicates were used for each group ^11^ and they were randomly selected to be assigned to the control and treatment groups. ^9^

We administered a single dose of 7,12-dimethylbenz[a]anthracene (DMBA), 20 mg/kg body weight, to induce mammary tumours in rats in INT and IT groups. The administration of DMBA was conducted twice weekly for five weeks. This carcinogenic agent has been widely used in many mammary tumour studies using SD rats. ^16^^,^^17^ Furthermore, the rats in the NIT and IT groups were treated with exposure to intermediate frequency (100 kHz) and low-intensity (18 Vpp) electric fields for 10 hours daily for 21 days in modified individual cages. ^9^ Mammary tumours were palpated every two days with a digital caliper and their size (cm ^2^) was tabulated. Nodule size were not measured in volume due to tool limitations. All tumour measurements were performed by the same investigator (NF). The therapy was terminated once the mammary tumours increased to 2.25 cm ^2^ in size or therapy was completed on day 21. All rats were returned to their communal cages every day after therapy had completed.

Individual cages were cleaned daily by removing rat droppings and changing feed and water. ^9^ Rat fur was given picric acid as an individual marker to avoid potential confounders, while rat cages were labeled with paint markers as group markers. Each work in this study, such as DMBA administration, euthanised rat dissection, kidney and liver sample fixation and data analysis, was carried out by different investigators. One investigator (FA) controlled and monitored all works in this study.

### Necropsy and organ harvesting

After completion of the treatment, all animals were euthanised under anaesthesia using an overdose of ketamine (150 mg/kg of body weight) via intramuscular injection. The animals were dissected ventrally side up on a dissected box by the same surgeon (AGF). ^9^ Two kidneys and two liver organs from different rats were collected randomly from each group. These 16 organs were used for histological examination. The number of samples used for histopathological examination was representative.

### Renal histopathological analysis

Samples of the left kidney were taken from all groups by necropsy, washed using physiological saline (0.9% NaCl) and then fixed using 10% neutral buffered formalin (NBF). This organ was prepared for histopathological cross-sections using the paraffin method and hematoxylin and eosin (H&E) staining with a slightly modified protocol adapted from Bancroft and Cook. ^18^ A piece of the organ that has been fixed then dehydrated using ethanol with a grade of 70%, 80%, 90%, and 100% for 2-3 repetitions, then followed by 4 hours clearing process with xylol at room temperature. Furthermore, the organ was infiltrated by putting it in the liquid paraffin at 60 ^o^C for 50 minutes with 3 repetitions. The next step was embedding which is putting the organ in a paraffin mold that contain liquid paraffin, then cooling it at room temperature. Then paraffin block which contains the organ was sectioned with 4-5 μm thickness. Then the organ slices were placed on a glass slide and deparaffinized by dipping them in xylol for 3x5 minutes. Then dehydration was performed using graded alcohol 96%, 90%, 80%, 70%, 50%, and distilled water for 1 minute each. The slides were then dipped in a solution of hematoxylin dye for 2-5 minutes, and dehydrated with 50% and 70% alcohol. Furthermore, it was dipped in eosin dye for 5-10 minutes, and dehydrated with 70%, 80%, 90%, and 96% alcohol. The last step was clearing for 15 minutes in xylol, and finally covered the slide using a cover glass.

Histopathological scoring of the kidneys was performed using the post-examination masking method combined with the ordinal scoring method. ^19^ The scoring referred to the endothelial-glomerular-tubular-interstitial (EGTI) system ^20^ that adjusted to the research requirements by replacing the endothelial parameter with the number of congestions ( Table 1). The scoring was performed on the renal cortex and medulla in 100 fields per group with 40x objective lens magnification. Microphotographs were taken using Leica DM750 photomicrographic microscope. Kidney sample fixation and histopathological analysis were performed by the same researcher (NF).

**Table 1.** Histopathological scoring system for the kidney.

Tissue type	Injury	Score
Glomerular	No damage	0
	Thickening of Bowman capsule	1
	Retraction of glomerular tuft	2
	Glomerular fibrosis	3
Tubular	No damage	0
	Reversible damage	1
	Reversible damage with necrosis in tissue less than 25%	2
	Reversible damage with necrosis in tissue between 25% and 50%	3
	Reversible damage with necrosis in tissue more than 50%	4
Interstitial	No damage	0
	Inflammation or haemorrhage exists	1
	Inflammation or haemorrhage exists with necrosis in tissue less than 25%	2
	Inflammation or haemorrhage exists with necrosis in tissue between 25% and 60%	3
	Inflammation or haemorrhage exists with necrosis in tissue more than 60%	4
Congestion	No congestion	0
	Congestion in tissue less than 25%	1
	Congestion in tissue between 25% and 50%	2
	Congestion in tissue between 51% and 75%	3
	Congestion in tissue between 76% and 100%	4

### Liver histopathological analysis

The liver was washed in physiological saline (0.9% NaCl) and immersed in a fixative solution (10% NBF). The histological preparation of the liver was carried out using the paraffin method of haematoxylin and eosin staining following Bancroft and Cook ^18^ with the same steps as for kidney preparation. Histopathological scoring was performed using the ordinal post-examination masking method. Scoring was carried out in 100 fields per group using 40x objective lens magnification. Three parameters of damage, namely cellular damage, haemorrhage, and congestion were determined for the histopathological scoring system ^21^^–^^23^ ( Table 2). Liver sample fixation and histopathological analysis were performed by the same researcher (SEDN).

**Table 2.** Histopathological scoring system for the liver.

Tissue type	Injury	Score
Cellular damage	No damage	0
	Reversible damage with necrosis in tissue less than 15%	1
	Reversible damage with necrosis in tissue between 15% and 40%	2
	Reversible damage with necrosis in tissues between 41% and 70%	3
	Reversible damage with necrosis in tissue between 71% and 100%	4
Haemorrhagic	No damage	0
	<15%	1
	15–40%	2
	41–70%	3
	71–100%	4
Congestion	No congestion	0
	Congestion in tissue less than 15%	1
	Congestion in tissue between 15% and 40%	2
	Congestion in tissue between 41% and 70%	3
	Congestion in tissue between 71% and 100%	4

### Data analysis

All measured data were analysed using the appropriate methods and without any exclusion. Data were analysed qualitatively and quantitatively. Qualitative data analysis was carried out descriptively. For quantitative data analysis, normality test was carried out first using the Shapiro-Wilk test (α=0.05). The scoring results were then analysed statistically using the Kruskal-Wallis test, which was followed by the Mann-Whitney test (α=0.05), since the data were not normally distributed, to find significant differences among the group (p<0.05). All data were analysed statistically using the SPSS version 16 (RRID:SCR_002865) program by the same researcher (NF).

## Results

The outcome of this research was the comparison of the histological characteristics of the kidney and liver under exposure to non-contact electric fields, which will be explained coherently in the sections below.

### Histopathology of kidney

**Figure 1.**
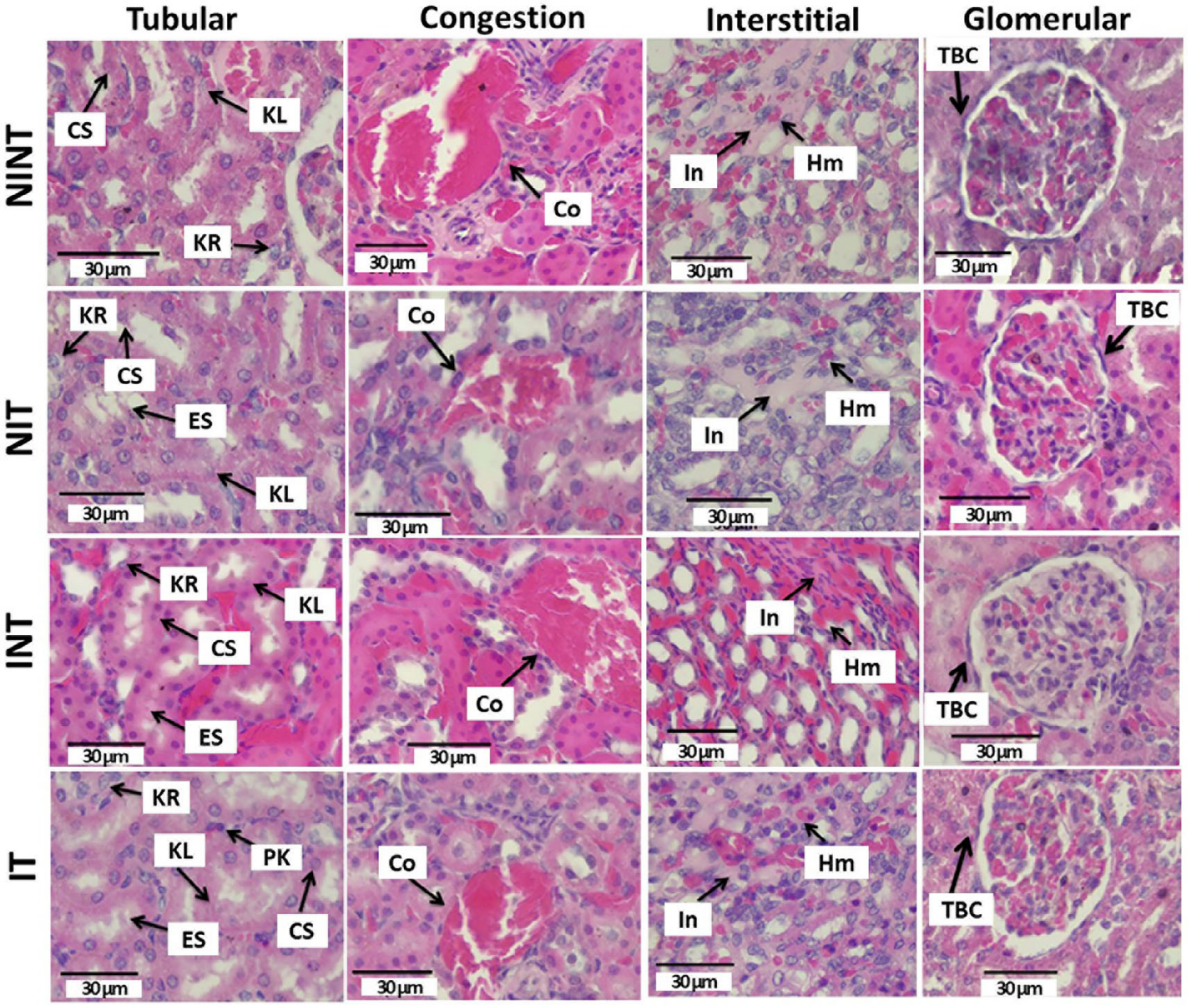
Histological features of tubular, interstitial, glomerular, and congestion damages in rat renal sections with H&E staining. KL=Karyolysis, KR=karyorrexhis, PK=pyknosis, CS=cloudy swelling, ES=epithelial sloughing, Co=congestion, In=inflammation, Hm=haemorrhage, TBC=thickening of Bowman’s capsule, NINT=non-induction and non-therapy group, NIT=non-induction and therapy group, INT=induction and non-therapy group, and IT=induction and therapy group.

**Figure 2.**
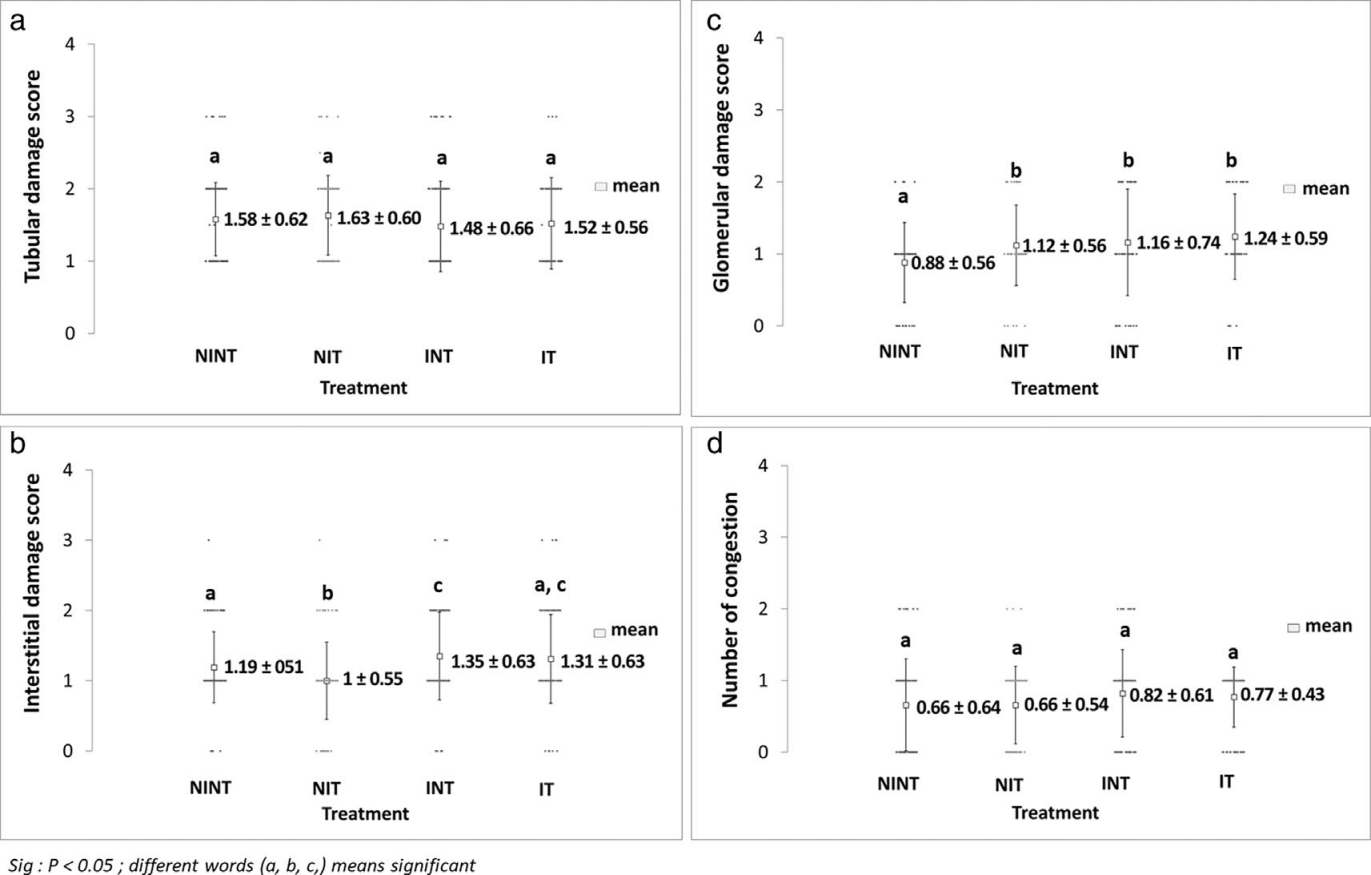
Scoring of tubular, interstitial, glomerular, and congestion damages in rat renal sections. (a) Tubular damages, (b) interstitial damages, (c) glomerular damages, and (d) number of congestions.

The effects of exposure to non-contact electric fields on renal histopathology and the ordinal scoring results of kidney damages are illustrated in Figure 1 and Figure 2, respectively. The main damage found in the kidney glomerular was the thickening of the Bowman capsule whose scores were significant in all treatment groups (1.12±0.56 for NIT, 1.16±0.74 for INT, and 1.24±0.59 for IT groups) compared to the control (NINT) group (0.88±0.56). In the kidney tubules, more damages were found, including karyolysis, karyorrexhis, pyknosis, cloudy swelling, and epithelial sloughing. However, the scores of these injuries were not significantly different among groups. In the kidney interstitial tissues, inflammation and haemorrhage were identified and the score of both damages in the placebo (NIT) group was the lowest (1.0±0.55) and significantly different to three other groups (1.19±0.51for NINT, 1.35±0.63 for INT, and 1.31±0.63 for IT groups). Congestion was found as a common injury in all parts of the kidney structure, and the number of congestions in the kidney structure of the placebo (NIT) group was also the lowest among treatment groups, but were not significantly different from the three other groups.

### Histopathology of liver

**Figure 3.**
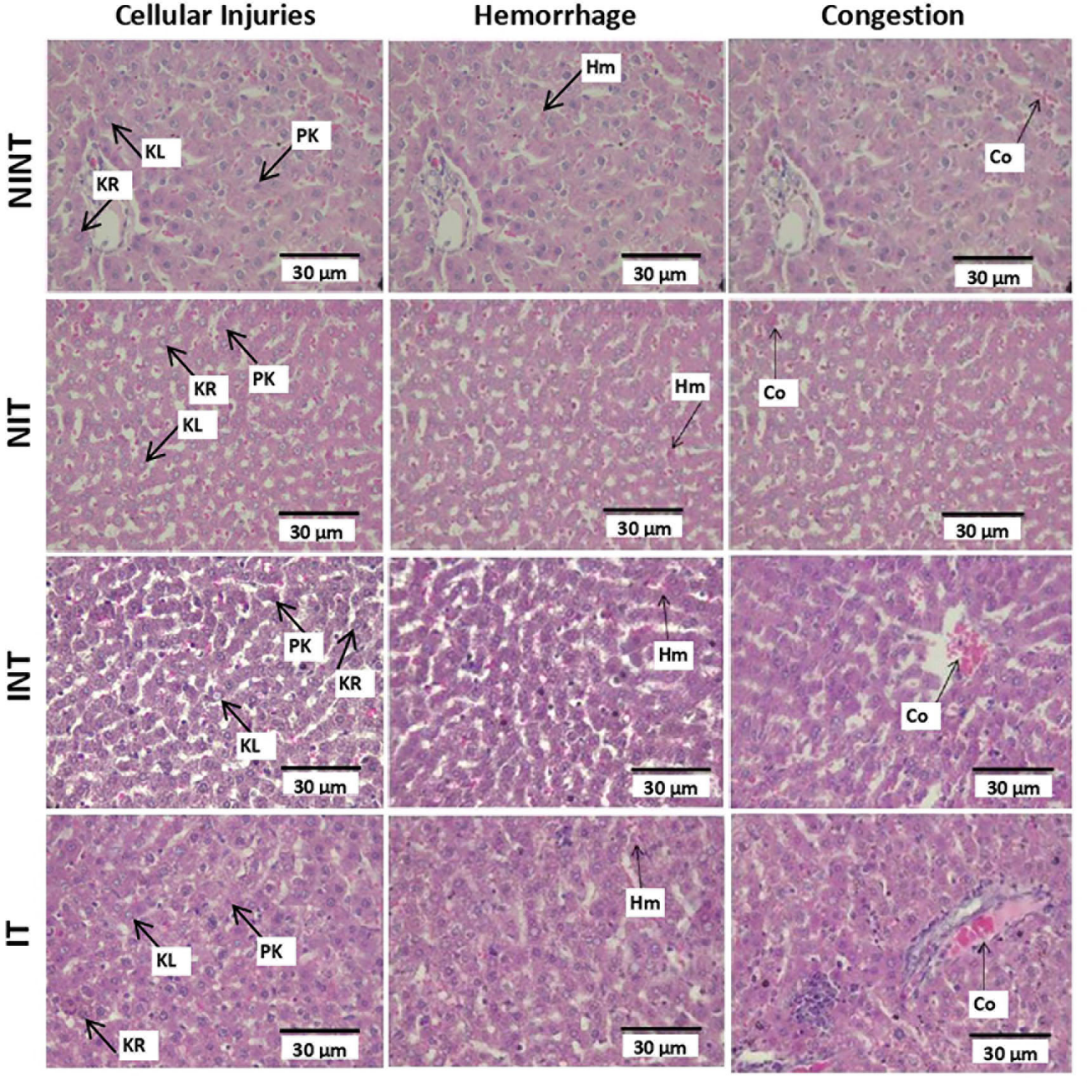
Histological features of haemorrhage, congestion, and cellular damages in rat liver section with H&E staining. Hr=Haemorrhage, Cg=congestion, Pn=pyknosis, Kr=karyorrhexis, Kl=karyolysis, Cs=cell swelling, Fc=fatty change, NINT=non-induction and non-therapy group, NIT=non-induction and therapy group, INT=induction and non-therapy group, and IT=induction and therapy group.

**Figure 4.**
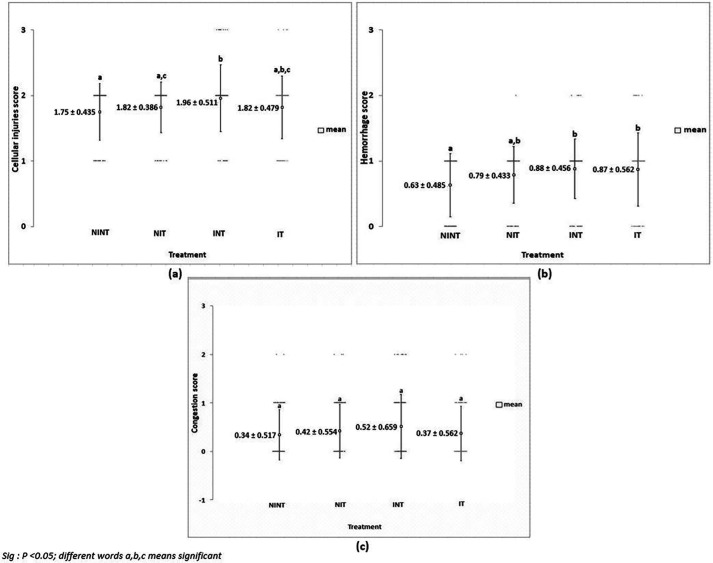
Scoring of cellular damages, haemorrhage, and congestion in rat liver sections. (a) Cellular damages, (b) haemorrhage, and (c) number of congestions.

The histopathological structure of the liver in the four groups had the same damage pattern but with different levels of damage as shown in Figure 3 and Figure 4. All groups had the same type of damage, namely cellular damage (pyknosis, karyolysis, karyorrhexis), haemorrhage and congestion, as well as reversible damage (cellular swelling and fatty change). No significant cellular damages were found after exposure to non-contact electric fields. Instead, the score of cellular injuries and hemorrhage was the highest after DMBA administration in INT group (1.96±0.51 and 0.88±0.46, respectively) and significantly different to control (NINT) group (1.75±0.43 and 0.63±0.48, respectively). The significant difference of hemorrhage score between IT group (0.87±0.56) and control (NINT) group (0.63±0.48),due to DMBA administration. Exposure to electric field in IT group slightly decreased hemorrhage, cellular injuries and congestion in liver (0.87±0.56, 1.82±0.48, 0.37±0.56, respectively) after DMBA administration as compared to INT group (0.88±0.46, 1.96±0.51, 0.52±0.66, respectively). The scores of congestion were also not significantly different among groups. The histology of the liver tissue in all groups did not show fibrosis, so it can be said that the congestion that occurred was not at a chronic level. Since there was no significant difference in the score of congestion among groups and fibrosis were not found, the congestion in all groups was still considered normal.

## Discussion

In the present study, the safety of the non-contact electric fields was revealed in the results of the histopathological analysis of kidney and liver in mammary tumour-induced rats, as discussed below.

The thickening of Bowman’s capsule as the main damage in the glomerulus ( Figure 1) may be a result of glomerular hyperfiltration, ^24^ nephrotoxicity caused by DMBA administration, ^25^ and exposure to electric fields. ^26^ Since the significant damages of the glomerulus were observed in the kidneys of the placebo (NIT), non-therapy (INT) and therapy (IT) groups, both DMBA administration and non-contact electric field exposure affected the thickening of Bowman’s capsule. Sharma and Paliwal ^27^ reported that kidneys are one of the major target organs of DMBA (nephrotoxicity) and that the epithelial cells of Bowman’s capsule and the proximal convoluted tubules appear to be more susceptible to DMBA. Whereas the electric fields affected this damage by changing the transmembrane potential and distribution of ion channels and dipoles following changes in the membrane structure. ^28^ Although both DMBA administration and exposure to non-contact electric fields affected the thickening of Bowman’s capsule, the individual toxicity effect of the electric field exposure was lower than the one with DMBA administration, as shown in Figure 2. Additionally, some biological effects of exposure to electric fields (0.6 and 340 kV/m) were revealed in humans and vertebrates, but no histological abnormalities were found in the organs, including the kidneys. ^29^ Therefore, DMBA administration would have a greater risk of inducing renal impairment.

The nephrotoxicity effect of DMBA did not occur only in the glomerulus, but also in the tubules. Moreover, DMBA caused substantive nephrotoxicity that is depicted by renal tubular necrosis including karyolysis, karyorrexhis and pyknosis, ^30^ as shown in Figure 1. Additionally, DMBA created obvious reversible histological changes in the tubules, such as epithelial sloughing and cloudy swelling, as illustrated in Figure 1. The epithelial sloughing represented the progressive disintegration of the tubules, ^29^ and the cloudy swelling may lead to cell necrosis. ^4^ However, since the score of each injury in the renal tubules was not significantly different among the groups, the nephrotoxicity effect of DMBA and the exposure to non-contact electric fields were not harmful to renal tubules. No reports have revealed necrosis or reversible injuries in renal tubules under exposure to the intermediate frequency and low-intensity electric fields, except exposure to 100 electric pulses resulting in high-intensity 575±67 V/cm electric fields for irreversible electroporation. ^31^ Therefore, the exposure to intermediate frequency and low intensity non-contact electric fields was not harmful to the renal tubules.

In renal interstitial tissues, the nephrotoxicity effect of DMBA significantly caused inflammation and haemorrhage, as shown in Figure 2. This inflammation can be affected by oxidative stress and may induce renal function impairment, including endothelial dysfunction, atherosclerosis, and glomerular injury. ^32^ Oxidative stress activates transcription factors including NF-kB, which activate the inflammatory response gene expression. ^33^ Moreover, Kandeel *et al.* ^34^ reported that oxidative stress may change the renal structure and function due to the effect of reactive oxygen species (ROS) on mesangial and endothelial cells. Oxidative injury happens when ROS, including O _2_, H _2_O _2_ and -OH, ruin the antioxidant defence systems of the cells. ^35^ This ROS may be produced due to DMBA administration ^36^ and it can spread from the site of production to other sites inside the cells or even extend the injury outside the cells. ^37^ Additionally, de Oliveira *et al.* ^38^ revealed that DMBA administration to develop a tumour in an animal model also causes haemorrhage. No reports have revealed inflammation and haemorrhage as well as congestion under exposure to intermediate frequency and low-intensity electric fields. Therefore, the exposure to non-contact electric fields was also not harmful to renal interstitial tissues. In fact, non-contact electric fields decreased the number of inflammations and haemorrhages in the placebo (NIT) group, as shown in Figure 2.

Almost the same as in kidney histology, there was no significant damage to the liver after exposure to electric fields ( Figure 4). The results in the non-therapy (INT) group with the highest significant rate of hepatocellular damage and hemorrhage indicated that DMBA as a carcinogenic substance can increase the presence of intercellular haemorrhage in the hepatic tissue. ^23^ Duarte *et al*. ^23^ reported mild hepatotoxicity in the liver, including the presence of a pyknotic phase of nuclei of hepatocytes due to DMBA induction. However, haemorrhage in the hepatic tissue has not yet shown symptoms of acute haemorrhage, including cellular hypoxia, decreased tissue perfusion, organ damage, and death. ^39^ The results in the therapy (IT) group with a lower rate of hepatocellular damage compared to the non-therapy (INT) one suggested that exposure to non-contact electric fields had lesser damaging effects than DMBA administration. Additionally, since the value of the congestion of blood vessels was still within the normal condition and not at the chronic level, the exposure to non-contact electric fields was not harmful. It was found that the intensity, frequency, and duration of exposure to non-contact electric fields and the dose of DMBA administered causes changes in the different parameters evaluated, although not very significant ones. However, these changes could be considered as a sign of metabolic alterations under the effect of the exposure to the non-contact electric fields and DMBA administration.

Based on the evidence for the efficacy and safety of normal tissues and organs, ^8^^,^^9^ including kidney and liver as reported in this study, we will be conducting a phase I clinical trial of ECCT for healthy volunteers using a 100 kHz 18 Vpp electric field as used in this study. Moreover, since this electric fields exposure may decrease the number of inflammations and haemorrhage in kidney, this therapy may be used to treat kidney injury or related diseases. ^40^

## Conclusions

The non-contact electric fields were not harmful to the renal and liver structure of tumour-induced rats. Instead, it may optimise the renal function in normal rats.

## Ethical approval

This research was carried out at the LPPT UGM and at the Animal Structure and Development Laboratory of the Faculty of Biology, UGM. LPPT UGM has been awarded ISO/IEC 17025:2000 accreditation for competence in testing and calibration. ^11^ Experimental protocol in this research was performed following approval by the Ethical Clearance Committee of LPPT UGM with ethical clearance number: 00015/4/LPPT/IV/2017, that has been previously reported. ^9^ The Ethical Clearance Committee stated that this research met the ethical requirements for the study on experimental animals and that the Ethical Clearance Committee had the right to conduct monitoring during the research.

## Data Availability

Open Science Framework: Kidney and liver histology in tumour-induced rats exposed to non-contact electric fields, https://doi.org/10.17605/OSF.IO/54BYF. ^41^ This project contains the following underlying data:
‐ Kidney and liver histological images ‐ Kidney scoring and statistical analysis ‐ Liver scoring and statistical analysis ‐ Kidney and liver charts Open Science Framework: Kidney and liver histology in tumour-induced rats exposed to non-contact electric fields, https://doi.org/10.17605/OSF.IO/54BYF. ^41^ This project contains the following extended data:
‐ Ethical clearance document Open Science Framework: ARRIVE checklist for ‘Kidney and liver histology in tumour-induced rats exposed to non-contact electric fields’, https://doi.org/10.17605/OSF.IO/54BYF. ^41^ Data are available under the terms of the Creative Commons Zero “No rights reserved” data waiver (CC0 1.0 Public domain dedication).
