# Effects of non-contact electric fields on kidney and liver histology in tumour-induced rats

**DOI:** 10.12688/f1000research.110080.3

**Published:** 2023-10-26

**Authors:** Firman Alamsyah, Nisrina Firdausi, Subekti Evi Dwi Nugraheni, Ahmad Ghitha Fadhlurrahman, Luthfi Nurhidayat, Rarastoeti Pratiwi, Warsito Purwo Taruno

**Affiliations:** 1 Faculty of Science and Technology, Universitas Al-Azhar Indonesia, Jl. Sisingamangaraja, Jakarta, 12110, Indonesia; 2 Center for Medical Physics and Cancer Research, Ctech Labs Edwar Technology, Tangerang, Banten, 15143, Indonesia; 3 Research Center for Pharmaceutical Ingridients and Traditional Medicine, National Research and Innovation Agency Republic of Indonesia, Jl. Raya Jakarta-Bogor Km. 46, Cibinong West Java, 16911, Indonesia; 4 Medical Laboratory, Universitas Pendidikan Indonesia, Jl. Dr. Setiabudhi No. 229, Bandung West Java, 40154, Indonesia; 5 Faculty of Biology, Universitas Gadjah Mada, Sleman, DI Yogyakarta, 55281, Indonesia

**Keywords:** damages, histology, kidney, liver, non-contact electric field, ECCT

## Abstract

**Background:** There were an estimated 2.1 million breast cancer diagnoses in 2018 worldwide, which is about 11.6% of the total cancer incidence. A novel modality of cancer treatment based on exposure to non-contact electric fields has been developed to reduce cancer incidence. However, the safety of the electric field exposure was not fully investigated. Therefore, the purpose of this study is to observe the safety of the electric field exposure on renal and liver structure.

**Methods:** Female Sprague-Dawley rats were divided into one control group and three treatment groups. Animals were treated with 7,12-dimethylbenz[a]anthracene for mammary tumour induction and exposed to non-contact electric fields individually for 10 hours a day for three weeks. Fresh samples of the kidney and liver were collected for observing structural damage in both organs. The two organs were prepared for histopathological cross-sectioning using the paraffin method and Hematoxylin & Eosin staining followed by histological scoring using the post-examination masking method.

**Results:** The damages found in the kidney were the following: thickening of Bowman capsule, karyolysis, karyorrexhis, pyknosis, cloudy swelling, epithelial sloughing, inflammation, haemorrhage, and congestion. The number of inflammation and haemorrhage in the kidney structure of the placebo group was the lowest and significantly different from the three other groups. All damages in the kidney were also found in the liver, but each showed different levels of damage. The damages in the kidney and liver caused by the exposure were not significant.

**Conclusions:** The non-contact electric fields were not harmful to renal and liver structure in tumour-induced rats. Instead, it may increase the renal function in normal rats.

## Introduction

The knowledge that electric fields can induce biological effects came to light in the 19 ^th^ century. Many studies have been conducted which provide evidence that exposure to electric fields can produce alterations in living things. ^1^ Several studies have examined the effects produced by electric fields on cell function. ^2^ Kirson *et al.* ^3^ reported that electric field intensity inside the cell is less than 10 V/cm, but inside the cell membrane, it can reach 10 ^5^ V/cm. At the organ level, the kidney and liver have dielectric properties that exhibit a time-temperature dependence. ^4^ ^–^ ^6^ Therefore, they possess both electrical conductivity and permittivity. ^5^ ^,^ ^6^

Porter *et al.* ^7^ explained that the knowledge of dielectric properties of biological tissues is invaluable and useful in several medical device applications, including cancer detection and treatment. For example, the cell proliferations of breast cancer, oral cancer, cervix cancer, osteosarcoma, and lung carcinoma, as well as intradermal melanoma and intracranial glioma, were successfully inhibited under exposure to intermediate frequency (100, 150, and 200 kHz) and low intensity (200 V/m) alternating electric fields with the duration of exposure to the electric fields for 24 to 72 hours for cell studies, and 10-12 hours per day for 14-21 days and 24 hours for 6 days. ^3^ ^,^ ^8^ ^–^ ^12^ Intermediate frequency electric fields are used to treat cancer because they specifically target cancer cells and do not affect normal cells due to their higher membrane potential than that of cancer cells. ^13^ ^,^ ^14^ In our preliminary study using 9 mice, the intermediate frequency (100 kHz) electric fields of Electro-Capacitive Cancer Therapy (ECCT) gave good results, wherein the tumour size was reduced by more than 67%, but showed no histological alterations in mammary and skin tissues. ^8^ We used a 100 kHz electric fields because this frequency gave the best results in our *in vitro* studies, where 28-39% of breast cancer cells died. ^8^ Furthermore, we developed non-contact electric fields to avoid dermatitis due to direct contact between the electrodes and the skin, as reported by Kirson *et al.* ^3^ This novel modality has the potential to reduce the global cancer burden; 2.1 million people around the world were diagnosed with breast cancer in 2018, which is 11.6% of the total cancer incidence. ^15^

Although non-contact electric fields-based therapy has the potential to treat cancer, the safety of such therapy in healthy tissues has to be investigated. This is because injury may occur after exposure to electric fields to organs such as the kidney and liver which have dielectric properties of the kidney and liver, which may interact with electric waves. Therefore, it is important to investigate abnormalities in the kidney and liver under exposure to electric fields during cancer treatment. The aim of this work was to investigate the safety of non-contact electric fields with a strength of 100 kHz-18 Vpp in the kidney and liver of animal tumour model, with a focus on possible histological alterations in the organs. We hypothesised that exposure to non-contact electric fields would not significantly affect the structure of the kidney and liver. According to our knowledge, this is the first study investigating the abnormalities in the kidney and liver under exposure to 100 kHz intermediate frequency and low-intensity (50-60 V/m) non-contact electric fields.

## Methods

### Experimental design

The experimental design and procedures, experimental animals, animal care and monitoring, housing and husbandry, sample size, inclusion and exclusion criteria, randomisation and blinding in this study were the same as our previously reported study. ^9^ For this study, 40 5-week-old healthy female Sprague Dawley (SD) rats ( *Rattus norvegicus,* Berkenhout 1769) weighing 50−80 g were used. This rat strain is one of the animals used as animal tumour models to study human breast cancer, since it has 98% genetic homology with humans. ^16^ These rats were provided by the Integrated Research and Testing Laboratory (LPPT) of Universitas Gadjah Mada (UGM), and have never been used for other studies. Rats that were sick or showing symptoms of disorder were excluded from the study. The rats were placed in polypropylene cages for one week of acclimatisation. The polypropylene cage used was communal cage with a size of 50 × 40 cm ^2^ and the base was covered with rice hull bedding. We prepared eight communal cages with each cage consisted of 5 animals. The lighting conditions in the animal’s room during the day came from lamp light, while at night it was total darkness (12L:12D photoperiod). We maintained room temperature to avoid dehydration during exposure to the electric field at 23–26°C with an average relative humidity of 81.09%.

We divided the animals into one control group (non-induction and non-therapy or NINT) and three treatment groups, namely placebo (non-induction and therapy or NIT), DMBA-induced mammary tumours without therapy (induction and non-therapy or INT), and DMBA-induced mammary tumours with therapy (induction and therapy or IT) group. Using Federer’s formula, the sample size in each group was calculated, in which 6 biological replicates were used for each group ^11^ and they were randomly selected to be assigned to the control and treatment groups. ^9^

**Figure 1.**
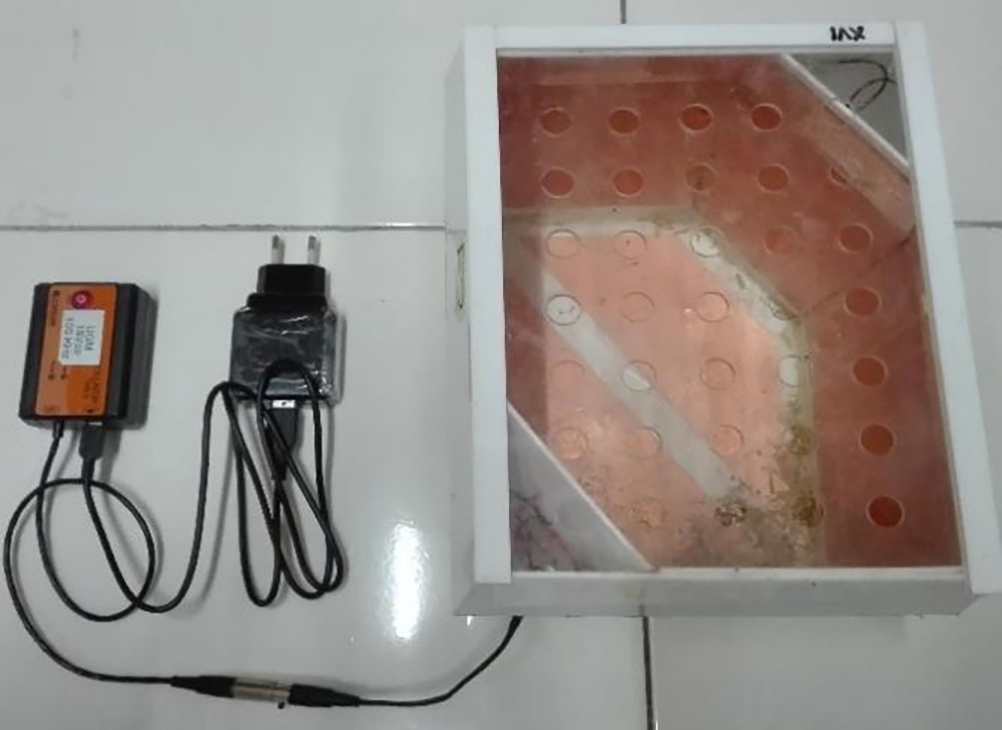
ECCT device for animal study. The size of the cage is 23 cm × 18 cm × 19 cm. The electrodes are attached to the acrylic wall of the cage with opposite polarity facing each other to produce multiple field directions.

We administered a single dose of 7,12-dimethylbenz[a]anthracene (DMBA), 20 mg/kg body weight, to induce mammary tumours in rats in the INT and IT groups. The administration of DMBA was conducted twice a week for five weeks. This carcinogenic agent has been widely used in many mammary tumour studies using SD rats. ^17^ ^,^ ^18^ Furthermore, the rats in the NIT and IT groups were treated with exposure to intermediate frequency (100 kHz) and low intensity (50-60 V/m) electric fields for 10 hours daily for 21 days in modified individual cages. ^9^ Alternating electric fields were generated between pairs of capacitive electrodes embedded in individual cages that have been modified into ECCT devices. ECCT is called non-contact because the electrodes do not stick directly to the animal’s skin. A multidirectional field was generated between pairs of capacitive electrodes and alternated every 0.5 ms ( Figure 1). All individual cages were placed on the same table at the same height. The experiment was carried out in a special room which only contained experimental animal cages. ^9^

The mammary tumour was palpated every two days with a digital caliper and its size (cm ^2^) was tabulated. Nodule size was not measured in volume due to tool limitation. All tumour measurements were performed by the same investigator (NF). The therapy was terminated once the mammary tumours enlarged to 2.25 cm ^2^ in size or therapy was completed on day 21. All rats were returned to their communal cages every day after the therapy was completed. Individual cages were cleaned daily by removing rat droppings and changing feed and water. ^9^ Rat fur was given picric acid as an individual marker to avoid potential confounders, while rat cages were labeled with a paint marker as a group marker. Each work in this study, such as DMBA administration, euthanised rat dissection, kidney and liver sample fixation, and data analysis, was carried out by a different investigator. One investigator (FA) controlled and monitored all works in this study.

### Necropsy and organ harvesting

After completion of the treatment, all animals were euthanised under anaesthesia using an overdose of ketamine (150 mg/kg of body weight) via intramuscular injection. The animals were dissected ventrally side up on a dissection box by the same surgeon (AGF). ^9^ Two kidneys and two livers from different rats were randomly collected from each group. A total of 16 organs were used for histological examination. The number of samples used for histopathological examination was quite representative.

### Renal histopathological analysis

Samples of the left kidney were taken from all groups by means of necropsy, washed with physiological saline (0.9% NaCl) and then fixed with 10% neutral buffered formalin (NBF). These organs were prepared for histopathological cross-sections using the paraffin method and hematoxylin and eosin (H&E) staining with a slightly modified protocol adapted from Bancroft and Cook. ^19^ A piece of organ that has been fixed was then dehydrated using graded ethanol 70%, 80%, 90%, and 100% for 2-3 repetitions, then followed by a 4 hours clearing process with xylol at room temperature. Furthermore, the organ was infiltrated by placing it in liquid paraffin at 60°C for 50 minutes with 3 repetitions. The next step was embedding, namely inserting the organ into a paraffin mold containing liquid paraffin, then cooling it to room temperature. Then the paraffin block containing the organ was cut 4-5 μm thick, and then the organ slices were placed on a glass slide and deparaffinized by dipping them in xylol for 3×5 minutes followed by dehydration using graded alcohol 96%, 90%, 80%, 70%, 50%, and distilled water for 1 minute each. The slides were then dipped in a hematoxylin dye solution for 2-5 minutes and dehydrated with 50% and 70% alcohol and subsequently dipped in eosin dye solution for 5-10 minutes, and dehydrated with 70%, 80%, 90%, and 96% graded alcohol. The last step was clearing in xylol for 15 minutes, and finally covered the slide with a cover glass.

Histopathological scoring of the kidneys was performed using the post-examination masking method combined with the ordinal scoring method. ^20^ The scoring referred to the endothelial-glomerular-tubular-interstitial (EGTI) system ^21^ which was adjusted to the needs of the study by replacing endothelial parameters with the number of congestion ( Table 1). The scoring was performed on the renal cortex and medulla at 100 visual fields per group with 40× objective lens magnification. Microphotographs were taken using a Leica DM750 photomicrographic microscope. Kidney sample fixation and histopathological analysis were performed by the same researcher (NF).

**Table 1.** Histopathological scoring system for the kidney.

Tissue type	Injury	Score
Glomerular	No damage	0
	Thickening of Bowman capsule	1
	Retraction of glomerular tuft	2
	Glomerular fibrosis	3
Tubular	No damage	0
	Reversible damage	1
	Reversible damage with necrosis in tissue less than 25%	2
	Reversible damage with necrosis in tissue between 25% and 50%	3
	Reversible damage with necrosis in tissue more than 50%	4
Interstitial	No damage	0
	Inflammation or haemorrhage exists	1
	Inflammation or haemorrhage exists with necrosis in tissue less than 25%	2
	Inflammation or haemorrhage exists with necrosis in tissue between 25% and 60%	3
	Inflammation or haemorrhage exists with necrosis in tissue more than 60%	4
Congestion	No congestion	0
	Congestion in tissue less than 25%	1
	Congestion in tissue between 25% and 50%	2
	Congestion in tissue between 51% and 75%	3
	Congestion in tissue between 76% and 100%	4

### Liver histopathological analysis

The liver was washed in physiological saline (0.9% NaCl) and immersed in a fixative solution (10% NBF). The histological preparations of the liver were carried out using the paraffin method, stained with haematoxylin and eosin following Bancroft and Cook ^19^ in the same steps as kidney preparations. Histopathological scoring was performed using the ordinal post-examination masking method. Scoring was carried out at 100 visual fields per group using a 40× objective lens magnification. Three parameters of damage, namely cellular damage, haemorrhage, and congestion were determined for the histopathological scoring system ^22^ ^–^ ^24^ ( Table 2). Liver sample fixation and histopathological analysis were performed by the same researcher (SEDN).

**Table 2.** Histopathological scoring system for the liver.

Tissue type	Injury	Score
Cellular damage	No damage	0
	Reversible damage with necrosis in tissue less than 15%	1
	Reversible damage with necrosis in tissue between 15% and 40%	2
	Reversible damage with necrosis in tissues between 41% and 70%	3
	Reversible damage with necrosis in tissue between 71% and 100%	4
Haemorrhagic	No damage	0
	<15%	1
	15–40%	2
	41–70%	3
	71–100%	4
Congestion	No congestion	0
	Congestion in tissue less than 15%	1
	Congestion in tissue between 15% and 40%	2
	Congestion in tissue between 41% and 70%	3
	Congestion in tissue between 71% and 100%	4

### Data analysis

All measured data were analysed using the appropriate methods and without any exclusion. Data were analysed qualitatively and quantitatively. Qualitative data analysis was carried out descriptively. For quantitative data analysis, the normality test was carried out first using the Shapiro-Wilk test (α=0.05). The scoring results were then analysed statistically to determine significant differences among groups (p<0.05) using the Kruskal-Wallis test followed by the Mann-Whitney test (α=0.05), since the data were not normally distributed. All data were statistically analysed using SPSS program version 16 (RRID:SCR_002865) by the same researcher (NF).

## Results

The result of this study is a comparison of the histological characteristics of the kidney and liver under exposure to non-contact electric fields, which will be coherently described in the sections below.

### Histopathology of kidney

**Figure 2.**
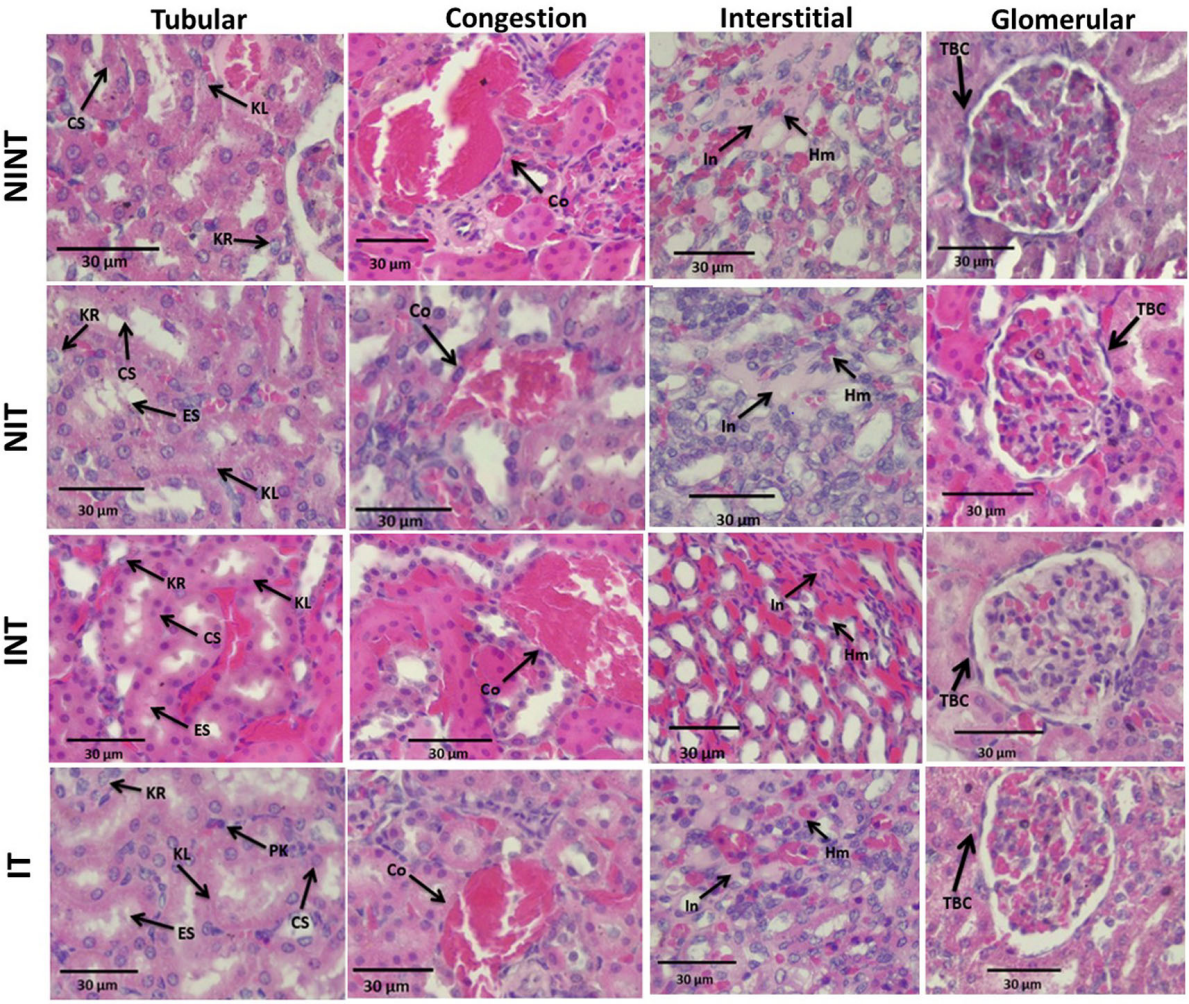
Histological features of tubular, interstitial, glomerular damages, and congestion in rat kidney sections stained with H&E. KL=Karyolysis, KR=karyorrexhis, PK=pyknosis, CS=cloudy swelling, ES=epithelial sloughing, Co=congestion, In=inflammation, Hm=haemorrhage, TBC=thickening of Bowman’s capsule, NINT=non-induction and non-therapy group, NIT=non-induction and therapy group, INT=induction and non-therapy group, and IT=induction and therapy group.

**Figure 3.**
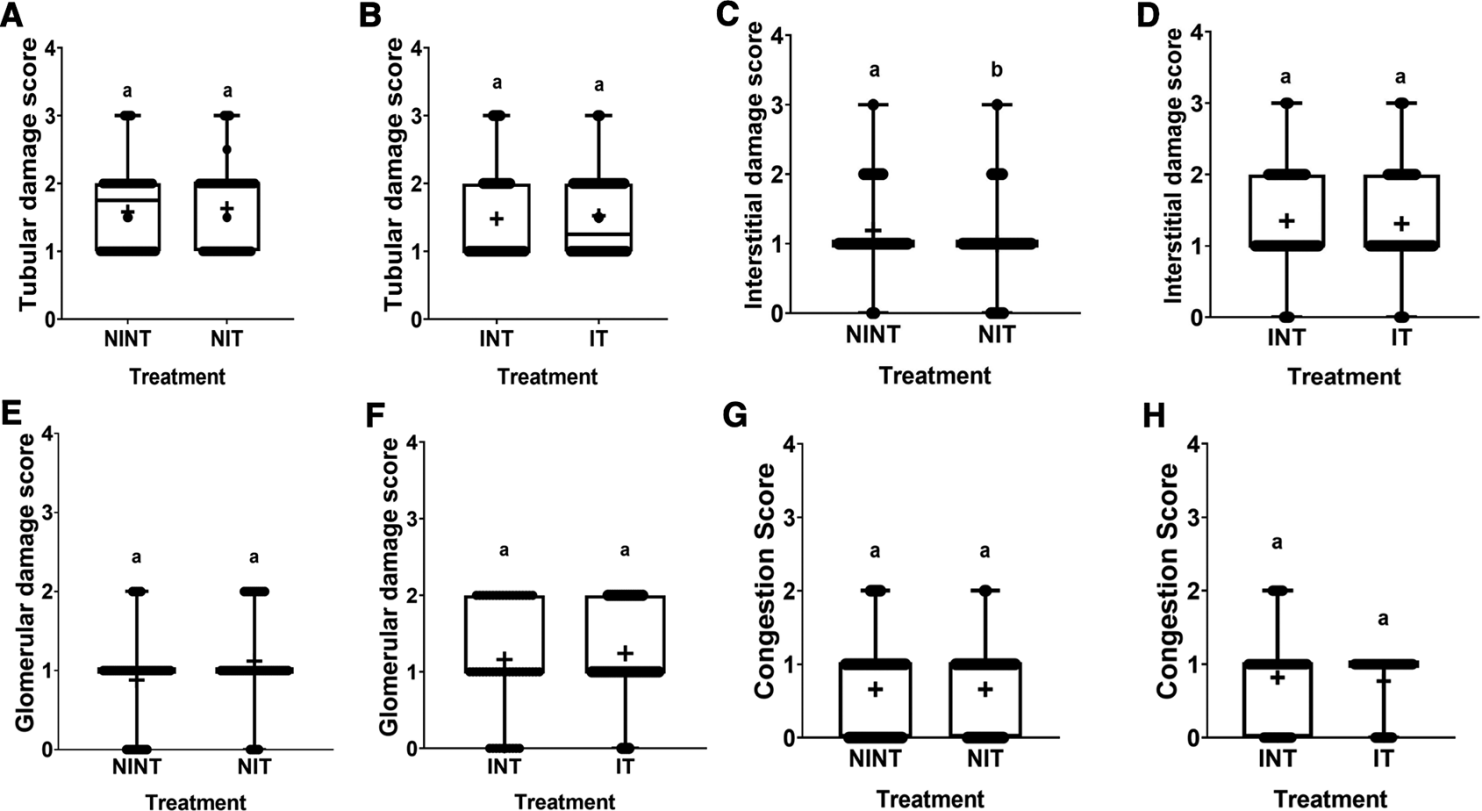
Scoring of tubular, interstitial, glomerular damages, and congestion in rat kidney sections. (A and B) Tubular damage, (C and D) interstitial damage, (E and F) glomerular damage, and (G and H) number of congestions.

The effects of non-contact electric fields exposure on renal histopathology and kidney damages scoring results are illustrated in Figure 2 and Figure 3, respectively. Some damages were found in the kidney tubules, including karyolysis, karyorrhexis, pyknosis, cloudy swelling, and epithelial sloughing. However, the damage scores were not significantly different (p>0.05), either in the kidneys of healthy rats ( Figure 3A) or in the kidneys of rats with breast cancer ( Figure 3B). In the renal interstitial tissues, inflammation and haemorrhage were identified. Figure 3C shows the score for both damages in the NIT group (1.0±0.55) was significantly lower (p<0.05) than that in the NINT group (1.19±0.51). The main damage found in the kidney glomerulus was thickening of Bowman’s capsule. However, non-contact electric field exposure did not cause significant glomerular damages (p>0.05) in the kidneys of healthy rats ( Figure 3E) or in the kidneys of rats with breast cancer ( Figure 3F). Congestion was found as a common injury in all parts of the kidney structure, and the number of congestions in the kidney structure. Similar to tubular and glomerular damages, and the number of congestions was not significantly different (p>0.05), either in the kidneys of healthy rats ( Figure 3G) or in the kidneys of rats with breast cancer ( Figure 3H).

### Histopathology of liver

**Figure 4.**
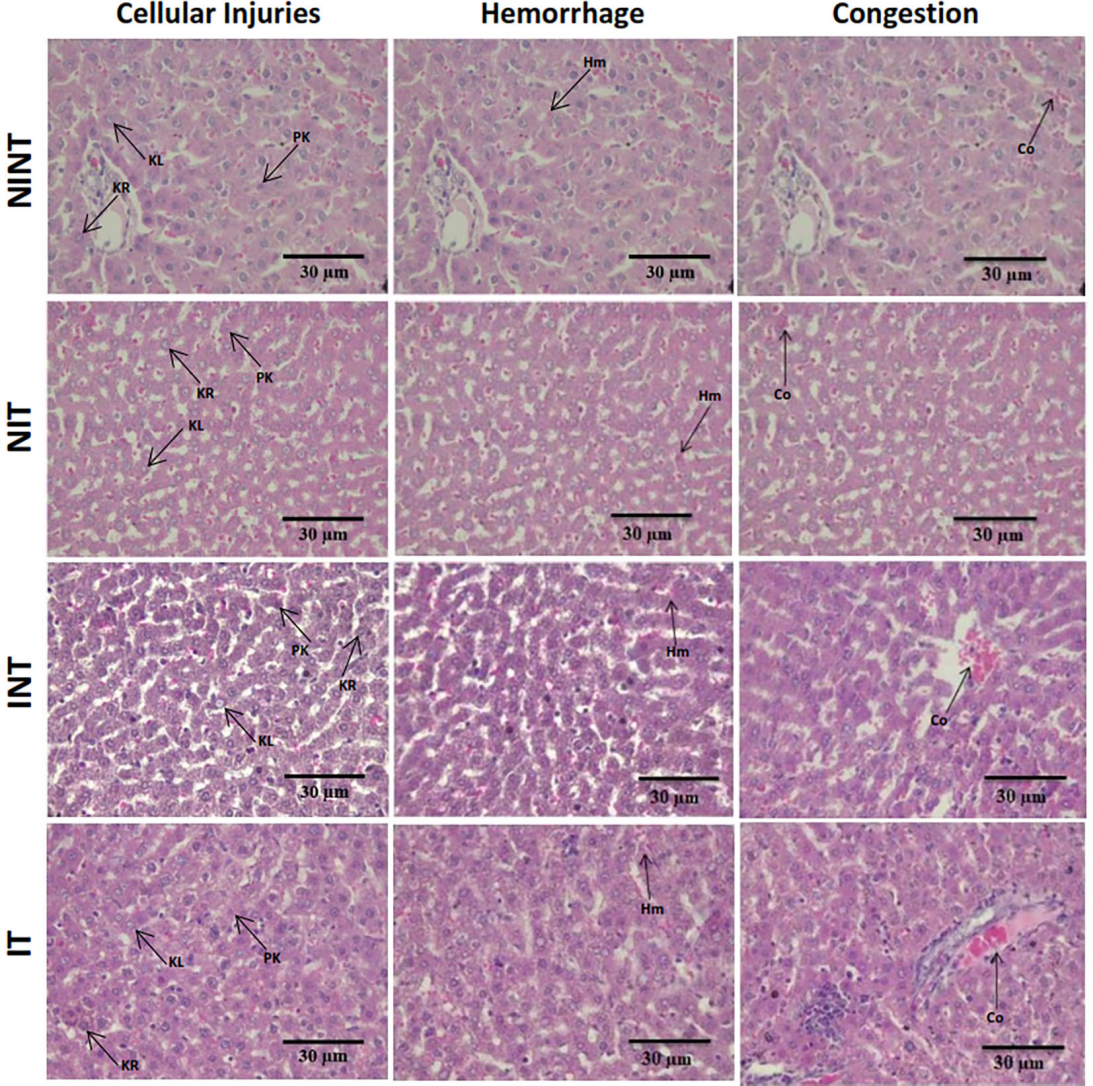
Histological features of haemorrhage, congestion, and cellular damage in rat liver sections stained with H&E. Hr=Haemorrhage, Cg=congestion, Pn=pyknosis, Kr=karyorrhexis, Kl=karyolysis, Cs=cell swelling, Fc=fatty change, NINT=non-induction and non-therapy group, NIT=non-induction and therapy group, INT=induction and non-therapy group, and IT=induction and therapy group.

**Figure 5.**
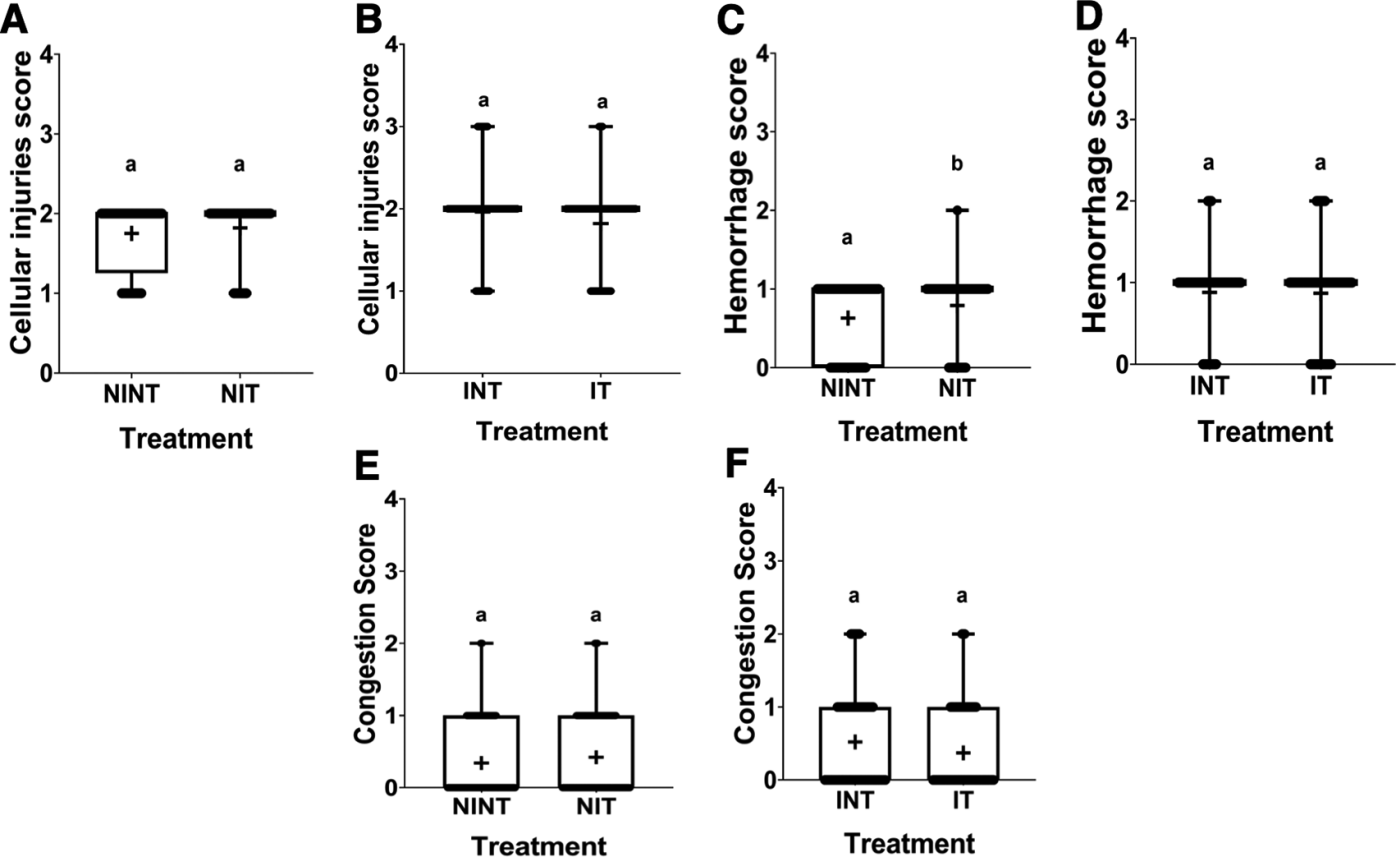
Scoring of cellular damage, haemorrhage, and congestion in rat liver sections. (A and B) Cellular damage, (C and D) haemorrhage, and (E and F) number of congestions.

The histopathological structure of the liver in the four groups had the same pattern of damage but with different levels of damage as shown in Figure 4 and Figure 5. All groups had the same type of damage, namely cellular damage (pyknosis, karyolysis, karyorrhexis), haemorrhage and congestion, and reversible damage (cellular swelling and fatty change). No significant cellular damage was found in the liver after exposure to non-contact intermediate frequency electric fields (p>0.05), either in the livers of healthy rats ( Figure 5A) or in the livers of rats with breast cancer ( Figure 5B). Figure 5C showed a significant difference in hemorrhage scores (p<0.05) in healthy rats livers between the NIT group (0.79±0.43) and the NINT group (0.63±0.48). The higher hemorrhage scores in the NIT group may indicate that liver cells that are also actively dividing are sensitive to intermediate frequency electric fields. However, there was no significant difference in hemorrhage scores (p>0.05) in the livers of rats with breast cancer between the IT and the INT groups ( Figure 5D). Exposure to intermediate frequency electric field in the IT group slightly decreased hemorrhage, cellular injury and congestion in the liver (0.87±0.56, 1.82±0.48, 0.37±0.56, respectively) after DMBA administration compared to the INT group (0.88±0.46, 1.96±0.51, 0.52±0.66, respectively). The scores of congestions also were not significantly different, either in the livers of healthy rats ( Figure 5E) or in the livers of rats with breast cancer ( Figure 5F). The histology of the liver tissue in all groups did not show any fibrosis, so it can be said that the congestion that occurred was not at a chronic level. Since there was no significant difference in the scores of congestions and no fibrosis was found, congestion in all groups was still considered normal.

## Discussion

In the present study, the safety of the non-contact intermediate frequency electric fields was revealed in the results of the histopathological analysis of kidney and liver in mammary tumour-induced rats, as discussed below.

The thickening of Bowman’s capsule as the main damage to the glomerulus ( Figure 2) may be a result of glomerular hyperfiltration, ^25^ DMBA-induced nephrotoxicity, ^26^ and exposure to electric fields. ^27^ Since there was no significant glomerular damage was observed in healthy rats (NIT group) and in rats with breast cancer (IT group) non-contact electric field exposure did not affect the thickening of Bowman’s capsule. Therefore, the electric fields exposure may not change the transmembrane potential and the distribution of ion channels and dipoles. ^28^ Similar results were also shown in our other study using an electric field frequency of 150 kHz with the same intensity (50-60 V/m), thickening of Bowman’s capsule was also found, but exposure to electric fields also did not significantly affect the damage. ^29^

The nephrotoxic effect of DMBA did not only occur in the glomerulus, but also in the tubules. In addition, DMBA caused substantive nephrotoxicity which is characterized by renal tubular necrosis including karyolysis, karyorrexhis and pyknosis, ^30^ as shown in Figure 2. Moreover, DMBA created obvious reversible histological changes in the tubules, such as epithelial sloughing and cloudy swelling, as illustrated in Figure 2. Epithelial sloughing represented the progressive tubular disintegration, ^31^ and cloudy swelling may lead to cell necrosis. ^4^ Conversely, electric fields exposure may direct the migration of mesenchymal stem cells to ameliorate acute nephrotoxicity ^32^ caused by DMBA. Therefore, exposure to electric fields did not increase tubular damage in the kidneys of rats with breast cancer (IT group) and the difference in damage was not significant compared with the untreated rats (INT group). In addition, exposure to non-contact electric field also did not cause significant tubular damage in healthy rats (NIT group), so exposure to this electric field was not harmful to the renal tubules. In our other study using an electric field frequency of 150 kHz with the same intensity (50-60 V/m), DMBA induction significantly damaged renal tubules. This suggests a nephrotoxic effect of DMBA to the renal tubules. In contrast, exposure to this electric field frequency resulted in a decrease in tubular damage in the normal kidneys (NIT group). Moreover, the frequency of this electric field may be able to compensate for tissue repair from the damage caused by DMBA. ^29^ Therefore, exposure to intermediate frequency and low intensity non-contact electric fields was not harmful to the renal tubules. It can even improve the condition of damaged renal tubules.

In the renal interstitial tissue, the nephrotoxic effect of DMBA caused inflammation and haemorrhage, as shown in Figure 2. This inflammation can be affected by oxidative stress and can lead to impaired kidney function, including endothelial dysfunction, atherosclerosis, and glomerular injury. ^33^ Oxidative stress activates transcription factors including NF-kB, which activates expression of inflammatory response gene. ^34^ In addition, Kandeel *et al.* ^35^ reported that oxidative stress may alter kidney structure and function due to the effects of reactive oxygen species (ROS) on mesangial and endothelial cells. Oxidative injury happens when ROS, including O _2_, H _2_O _2_ and -OH, ruin the antioxidant defence system of the cells. ^36^ These ROS can be produced due to DMBA administration ^37^ and can spread from their site of production to other sites inside the cell or even prolong the injury outside the cell. ^38^ Moreover, de Oliveira *et al.* ^39^ revealed that DMBA administration to develop tumours in animal models also causes haemorrhage. In contrast, exposure to this non-contact electric field significantly decreased the number of inflammations and haemorrhages in the healthy rats (NIT group), as shown in Figure 3C. To the best of our knowledge, these results are the first findings showing that non-contact electric fields exposure can reduce inflammation and hemorrhage in rat kidneys. In our other study using an electric field frequency of 150 kHz with the same intensity (50-60 V/m), interstitial injury was not significantly induced by exposure to electric fields. ^29^ In another study using electromagnetic field exposure to 150 kHz in healthy SD rats, showed normal kidney morphology, including normal-appearing glomeruli, tubules, and interstitium. ^40^ Therefore, exposure to non-contact intermediate frequency electric fields was also not harmful to the renal interstitial tissue.

In contrast to kidney histology, there was a significant damage, namely hemorrhage, in the liver of healthy rats (NIT group) after exposure to intermediate frequency non-contact electric fields ( Figure 5C). Meanwhile, in rats with breast cancer (IT group) there was no significant hemorrhagic damage (Figure 5D). Liver cells are cells that actively divide and have a membrane potential that is the same as the membrane potential of breast cancer cells. ^13^ ^,^ ^14^ With these characteristics, liver cells can be sensitive to exposure to non-contact electric fields. However, haemorrhage in the hepatic tissue has not shown symptoms of acute haemorrhage, such as cellular hypoxia, decreased tissue perfusion, organ damage, and death. ^41^ In addition, different results were obtained in our other study using an electric field frequency of 150 kHz. Exposure to this electric field can significantly reduce haemorrhage in the liver of both healthy rats (NIT group) and tumour-induced rats (IT group). ^29^ In another study using exposure to a 150 kHz electromagnetic field, showed mild inflammatory changes with lymphocytic infiltration and haemorrhages in the liver of healthy rats indicating possible liver damage or infection. However, the liver damage that occurred was insufficient to cause clinical and functional manifestations because the lesions were mild enough without significant changes in liver enzyme levels. ^40^

The results in the therapy (IT) group with a lower hepatocellular damage score compared to the non-therapy (INT) group suggested that exposure to non-contact electric fields was not harmful to the livers of rats with breast cancer, even tends to repair the hepatocellular damage. In addition, since the vascular congestion score was still in a normal condition and not at a chronic level, exposure to non-contact electric fields was not harmful. In our other study using an electric field frequency of 150 kHz with the same intensity (50-60 V/m), hepatocellular damage and congestion were not significantly induced by exposure to electric fields. ^29^ Therefore, exposure to intermediate frequency non-contact electric fields was not harmful to the livers of the animals.

Damage to the kidneys and liver of the rats in the control group (NINT) cannot be predicted because rats with symptoms of illness had been excluded and rats were also randomly selected for each group. The thickening of Bowman’s capsule in the NINT group can occur naturally due to aging, or due to ischemia. ^29^ ^,^ ^42^ Injury to normal renal tubules can occur because of the high rate of reabsorption by the renal tubules. ^29^ For damage to the renal interstitial tissue, a score below 2 indicates that there is little inflammation or hemorrhage. Inflammation is part of the activation of the immune system in response to acute or chronic kidney injury which can be caused by pathogens that enter the rat’s body. ^43^ For damage to the liver in the NINT group, if we look at the hemorrhagic and congestion scores which are below 1, this indicates that there is little or no damage to the liver. For a cellular damage score below 2, this indicates reversible damage with less than 15% necrosis. Liver hepatocytes have many vital functions, so they can proliferate extensively, which allows efficient regeneration of the liver for reversible damage. ^44^ In addition, the liver itself is a very vulnerable organ due to its size and is the organ most frequently injured after abdominal trauma. ^45^

For the results of this study, we only reported the effect of the intermediate frequency non-contact electric field on the histological structure of the kidney and liver, not yet on their function. Renal function parameters such as creatinine and bilirubin, and liver function parameters such as aspartate aminotransferase (AST) and alanine transaminase (ALT) taken from blood serum samples will be reported together with the hematological profile of the rat blood. Based on the evidence of the efficacy and safety of ECCT on normal tissues and organs, ^8^ ^,^ ^9^ ^,^ ^29^ including kidney and liver as reported in this study, we will conduct a phase I clinical trial of ECCT for healthy volunteers using an intermediate frequency (100 kHz) electric field as used in this study. Moreover, since this electric field exposure can reduce the number of inflammations and haemorrhages in the kidneys, this therapy can be used to treat kidney injuries or related diseases. ^46^

## Conclusions

Exposure to a non-contact electric field with intermediate frequency had a variety of effects on kidney and liver tissues. Exposure to this electric field can cause hemorrhagic damage in livers of healthy rats, however, in other liver tissues and in the kidneys, exposure to this electric field was safe. In addition, exposure to this electric fields did not cause significant hemorrhagic damage in rats with breast cancer. It can even decrease the number of inflammations and haemorrhages in the kidneys.

### Ethical approval

This research was carried out at the LPPT UGM and at the Animal Structure and Development Laboratory of the Faculty of Biology, UGM. LPPT UGM has been awarded ISO/IEC 17025:2000 accreditation for competence in testing and calibration. ^11^ Experimental protocol in this research was performed following approval by the Ethical Clearance Committee of LPPT UGM with ethical clearance number: 00015/4/LPPT/IV/2017, that has been previously reported. ^9^ The Ethical Clearance Committee stated that this research met the ethical requirements for the study on experimental animals and that the Ethical Clearance Committee had the right to conduct monitoring during the research.

## Data availability

### Underlying data

Open Science Framework: Kidney and liver histology in tumour-induced rats exposed to non-contact electric fields, https://doi.org/10.17605/OSF.IO/54BYF. ^47^

This project contains the following underlying data:

• Kidney and liver histological images
• Kidney scoring and statistical analysis
• Liver scoring and statistical analysis
• Kidney and liver charts

### Extended data

Open Science Framework: Kidney and liver histology in tumour-induced rats exposed to non-contact electric fields, https://doi.org/10.17605/OSF.IO/54BYF. ^47^

This project contains the following extended data:

• Ethical clearance document

### Reporting guidelines

Open Science Framework: ARRIVE checklist for ‘Kidney and liver histology in tumour-induced rats exposed to non-contact electric fields’, https://doi.org/10.17605/OSF.IO/54BYF. ^47^

Data are available under the terms of the Creative Commons Zero “No rights reserved” data waiver (CC0 1.0 Public domain dedication).
