# Effects of non-contact electric fields on the kidneys and livers of tumour-bearing rats

**DOI:** 10.12688/f1000research.110080.6

**Published:** 2025-02-11

**Authors:** Firman Alamsyah, Nisrina Firdausi, Subekti Evi Dwi Nugraheni, Ahmad Ghitha Fadhlurrahman, Luthfi Nurhidayat, Rarastoeti Pratiwi, Warsito Purwo Taruno

**Affiliations:** 1 Faculty of Science and Technology, Universitas Al-Azhar Indonesia, Jl. Sisingamangaraja, Jakarta, 12110, Indonesia; 2 Center for Medical Physics and Cancer Research, Ctech Labs Edwar Technology, Tangerang, Banten, 15143, Indonesia; 3 Research Center for Pharmaceutical Ingridients and Traditional Medicine, National Research and Innovation Agency Republic of Indonesia, Jl. Raya Jakarta-Bogor Km. 46, Cibinong West Java, 16911, Indonesia; 4 Medical Laboratory, Universitas Pendidikan Indonesia, Jl. Dr. Setiabudhi No. 229, Bandung West Java, 40154, Indonesia; 5 Faculty of Biology, Universitas Gadjah Mada, Sleman, DI Yogyakarta, 55281, Indonesia

**Keywords:** damages, histology, kidney, liver, non-contact electric field, ECCT

## Abstract

**Background:**

A novel modality of cancer treatment based on exposure to non-contact electric fields called Electro-Capacitive Cancer Therapy (ECCT) has been developed. However, the effects of this modality on vital organs during cancer treatment have not been fully investigated. Therefore, this study aimed to investigate the effects of non-contact electric field exposure on kidney and liver structures.

**Methods:**

Female rats were randomly divided into one control group and three treatment groups with six replications each. Animals were treated with 7,12-dimethylbenz[a]anthracene at a dose of 20 mg/kg body weight for mammary tumour induction. Animals were then exposed to electric fields (100 kHz, 50-60 V/m) for 10 hours a day for three weeks. Two kidney samples and two liver samples from different animals in each group were collected for observation of structural damage to the organs. Histopathological cross-sections of the kidneys and livers were made using the paraffin method and Hematoxylin-Eosin staining. Histological scoring used the post-examination masking method with 100 visual fields per group.

**Results:**

There was no significant damages to the tubules, glomeruli, and interstitial of the kidneys, including congestion, after exposure to non-contact electric fields. In addition, healthy rats exposed to this electric field showed significantly lower renal interstitial damage. There was no significant cellular damage, congestion, and haemorrhage in the livers of all groups, except in the healthy rat group that showed significantly higher haemorrhage.

**Conclusions:**

Exposure to non-contact electric fields may cause haemorrhage in the livers of healthy rats. However, in kidney tissue, exposure to this electric field was tolerable, and can even decrease the number of inflammations and haemorrhages in healthy rats.

## Introduction

The knowledge that electric fields can induce biological effects came to light in the 19 ^th^ century. Many studies have been conducted which provide evidence that exposure to electric fields can produce alterations in living things. ^1^ For example, frequency-dependent electric fields can regulate intracellular signaling and cell function. ^2^ At the organ level, the kidney and liver have dielectric properties that exhibit a time-temperature dependence. ^3^ ^–^ ^6^ Therefore, they possess both electrical conductivity and permittivity. ^5^ ^,^ ^6^

Porter *et al.* ^7^ explained that the knowledge of dielectric properties of biological tissues is invaluable and useful in several medical device applications, including cancer detection and treatment. For example, the proliferation of some cancer cells was successfully inhibited under exposure to intermediate-frequency (100, 150, and 200 kHz) and low-intensity (200 V/m) alternating electric fields. The duration of electric field exposure was 24 to 72 hours for cell studies, and 10-12 hours per day for 14-21 days for animal studies ^3^ ^,^ ^8^ ^–^ ^12^ This range of electric fields frequencies are used to treat cancer because they specifically target cancer cells. In addition, they do not affect normal cells due to their higher membrane potential than that of cancer cells. ^13^ ^,^ ^14^ In our preliminary study using 9 mice, the 100 kHz frequency and 50-60 V/m intensity of electric fields of Electro-Capacitive Cancer Therapy (ECCT) gave good results. The results of the study showed that the tumour size was reduced by more than 67%, and showed no histological alterations in the breast and skin tissues. ^8^ In addition, further studies using tumour-bearing rats showed that exposure to a 100 kHz and 50-60 V/m electric field could significantly reduce the size of breast tumour nodules in rats (p<0.05) by up to 71.5%. ^9^ Meanwhile, other study using 150 kHz electric field exposure were unable to reduce the size of breast tumour nodules in rats, although cancer cell death occurred. ^11^ The electric fields with 100 kHz frequency and 50-60 V/m intensity also gave the best results in our *in vitro* studies, where 28-39% of breast cancer cells died. ^8^ The frequency of 100 kHz was also used to inhibit the growth rate of liver tumors by exposure to magnetic fields and this frequency did not affect the viability of normal liver cells. ^15^ Furthermore, we developed non-contact electric fields to avoid dermatitis due to direct contact between the electrodes and the skin. ^9^ This novel modality has the potential to reduce the global cancer burden; 2.1 million people around the world were diagnosed with breast cancer in 2018, which is 11.6% of the total cancer incidence. ^16^ In addition, non-contact electric field therapy has also been developed to treat chronic diabetic ulcers, ^17^ and to treat metastatic cancer. ^18^

Although non-contact electric field-based therapy has the potential to treat cancer, the effects of such therapy in healthy tissues has to be investigated. This is because injury may occur after exposure to electric fields in organs such as the kidney and liver which have dielectric properties. The dielectric properties of the kidneys and liver may interact with electric waves. Therefore, it is important to investigate abnormalities in the kidneys and liver under exposure to electric fields during cancer treatment. Nurhidayat *et al.* ^19^ reported the effects of non-contact electric field exposure with a frequency of 150 kHz on the kidneys and liver, and blood chemistry as well as a parameter to measure kidney and liver function. They demonstrated that exposure to 150 kHz non-contact electric fields did not significantly cause histopathological injury to the liver and kidneys. In addition, the exposure also did not harm the levels of creatinine, AST, and ALT in blood plasma. However, there are no reports on the effects of 100 kHz non-contact electric fields on the kidneys and liver of rats. Therefore, this work aimed to investigate the effects of non-contact electric fields with a strength of 100 kHz-50-60 V/m in the kidneys and liver of animal tumour models. The focus of this study was the possible histological alterations during exposure to electric fields in both organs. We hypothesised that exposure to non-contact electric fields of 100 kHz-50-60 V/m would not significantly affect the structure of the kidneys and liver, because this frequency belongs to the intermediate frequency as does the frequency of 150 kHz. According to our knowledge, this is the first study investigating the abnormalities in the kidneys and liver under exposure to intermediate-frequency (100 kHz) and low-intensity (50-60 V/m) non-contact electric fields.

## Methods

### Experimental design

The experimental design and procedures, experimental animals, animal care and monitoring, housing and husbandry, sample size, inclusion and exclusion criteria, randomisation, and blinding in this study were the same as our previously reported study. ^9^ For this study, 40 5-week-old healthy female Sprague Dawley (SD) rats ( *Rattus norvegicus,* Berkenhout 1769) weighing 50−80 g were used. This rat strain is one of the animals used as animal tumour models to study human breast cancer since it has 98% genetic homology with humans. ^20^ These rats were provided by the Integrated Research and Testing Laboratory (LPPT) of Universitas Gadjah Mada (UGM), and have never been used for other studies. Rats that were sick or showing symptoms of disorder were excluded from the study. The rats were placed in polypropylene cages for one week of acclimatisation. The polypropylene cage used was a communal cage with a size of 50 × 40 cm ^2^ and the base was covered with rice hull bedding. We prepared eight communal cages with each cage consisting of 5 animals. The lighting conditions in the animal’s room during the day came from lamp light, while at night it was total darkness (12L:12D photoperiod). We maintained room temperature to avoid dehydration during exposure to the electric field at 23–26°C with an average relative humidity of 81.09%.

We divided the animals into one control group (non-induction and non-therapy or NINT) and three treatment groups, namely placebo (non-induction and therapy or NIT), DMBA-induced mammary tumours without therapy (induction and non-therapy or INT), and DMBA-induced mammary tumours with therapy (induction and therapy or IT) group. Using Federer’s formula, the sample size in each group was calculated, in which 6 biological replicates were used for each group. ^11^ The animals were randomly selected to be assigned to the control and treatment groups. ^9^

**Figure 1.**
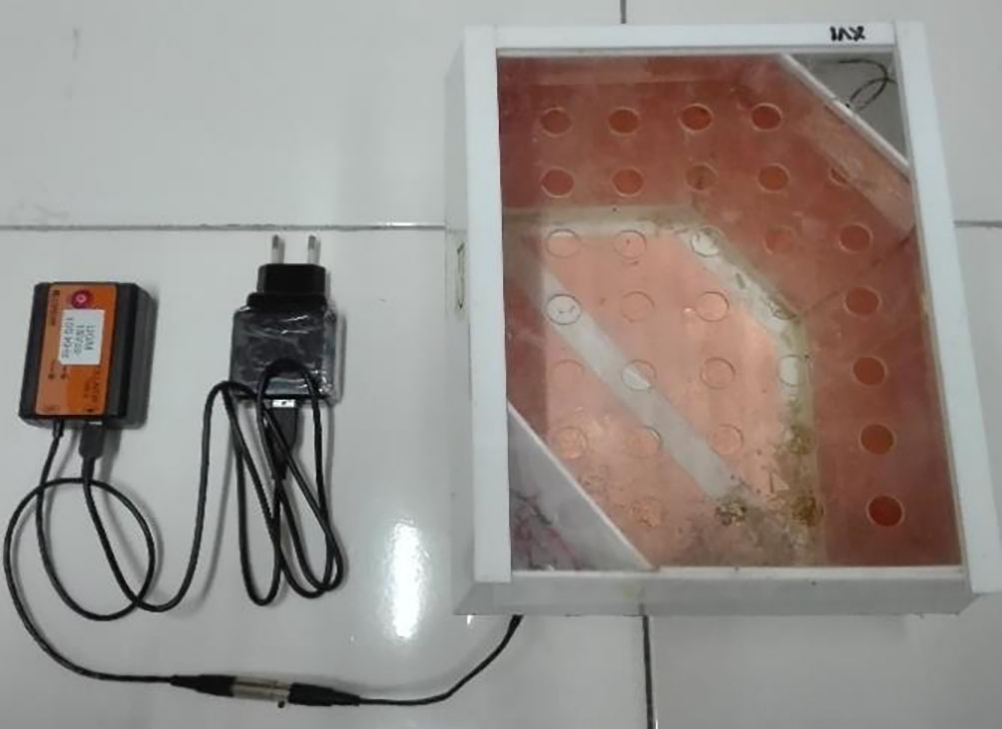
ECCT device for animal study. The size of the cage is 23 cm × 18 cm × 19 cm. The electrodes are attached to the acrylic wall of the cage with opposite polarity facing each other to produce multiple field directions.

We administered a single dose of 7,12-dimethylbenz [a] anthracene (DMBA), 20 mg/kg body weight, to induce mammary tumours in rats in the INT and IT groups. The administration of DMBA was conducted twice a week for five weeks. This carcinogenic agent has been widely used in many mammary tumour studies using SD rats. ^21^ ^,^ ^22^ Furthermore, the rats in the NIT and IT groups were treated with exposure to intermediate-frequency (100 kHz) and low-intensity (50-60 V/m) electric fields for 10 hours daily for 21 days in modified individual cages. ^9^ In preliminary study on mice, we used electric field exposure for 12 hours per day for 14 days. ^8^ We wanted to try to reduce the daily exposure in order to reduce the risk of animal stress, but with the result of reducing tumour nodules that remained good. Alternating electric fields were generated between pairs of capacitive electrodes embedded in individual cages that have been modified into ECCT devices. ECCT is called non-contact because the electrodes do not stick directly to the animal’s skin. A multidirectional field was generated between pairs of capacitive electrodes and alternated every 0.5 ms ( Figure 1). All individual cages were placed on the same table at the same height. The experiment was carried out in a special room that only contained experimental animal cages. ^9^

The mammary tumour was palpated every two days with a digital caliper and its size (cm ^2^) was tabulated. Nodule size was not measured in volume due to tool limitation. All tumour measurements were performed by the same investigator (NF). The therapy was terminated once the mammary tumours enlarged to 2.25 cm ^2^ in size or therapy was completed on day 21. All rats were returned to their communal cages every day after the therapy was completed. Individual cages were cleaned daily by removing rat droppings and changing feed and water. ^9^ Rat fur was given picric acid as an individual marker to avoid potential confounders. Rat cages were labeled with a paint marker as a group marker. Each work in this study, such as DMBA administration, euthanised rat dissection, kidney and liver sample fixation, and data analysis, was carried out by a different investigator. One investigator (FA) controlled and monitored all works in this study.

### Necropsy and organ harvesting

After completion of the treatment, all animals were euthanised under anaesthesia using an overdose of ketamine (150 mg/kg of body weight) via intramuscular injection. The animals were dissected ventrally side up on a dissection box by the same surgeon (AGF). ^9^ Two kidneys and two livers from different rats were randomly collected from each group. A total of 16 organs were used for histological examination. We only took 4 organs from 2 individual animals, and did not take organs from other individuals, because we considered the number of samples used for histological examination to be representative enough and had been approved by the ethics committee.

### Renal histopathological analysis

Samples of the left kidney were taken from all groups using necropsy, then washed with physiological saline (0.9% NaCl), and fixed with 10% neutral buffered formalin (NBF). These organs were prepared for histopathological cross-sections using the paraffin method. Hematoxylin and eosin (H&E) staining was performed with a slightly modified protocol adapted from Bancroft and Cook. ^23^ The fixed organ pieces were then dehydrated using graded ethanol of 70%, 80%, 90%, and 100% for 2-3 repetitions, and cleared for 4 hours with xylol at room temperature. Furthermore, the organ was infiltrated with liquid paraffin at 60°C for 50 minutes with 3 repetitions. The next step was embedding, where the organ is placed in a paraffin mold containing liquid paraffin, and then cooled to room temperature. Then the paraffin block containing the organ was cut 4-5 μm thick. Then the organ slices were placed on glass slides and deparaffinized in xylol for 3×5 minutes, and then dehydrated using graded alcohol of 96%, 90%, 80%, 70%, 50%, and distilled water for 1 minute each. Then the slides were dipped into hematoxylin dye solution for 2-5 minutes and dehydrated with 50% and 70% alcohol. The slides subsequently were dipped into eosin dye solution for 5-10 minutes, then dehydrated with graded alcohol of 70%, 80%, 90%, and 96%. The last step was clearing in Xylol for 15 minutes, and finally the slide was covered with a cover glass.

Histopathologic scoring of the kidneys was performed using the post-examination masking method combined with the ordinal scoring method. ^24^ The scoring referred to the endothelial-glomerular-tubular-interstitial (EGTI) system. ^25^ This EGTI system was adjusted to the needs of the study by replacing endothelial parameters with the number of congestion ( Table 1). The scoring was performed on the renal cortex and medulla in 100 fields of view per group at 40× objective lens magnification. Microphotographs were taken using a Leica DM750 photomicrographic microscope. Kidney sample fixation and histopathological analysis were performed by the same researcher (NF).

**Table 1.** Histopathological scoring system for the kidney.

Tissue type	Injury	Score
Glomerular	No damage	0
	Thickening of Bowman capsule	1
	Retraction of glomerular tuft	2
	Glomerular fibrosis	3
Tubular	No damage	0
	Reversible damage	1
	Reversible damage with necrosis in tissue less than 25%	2
	Reversible damage with necrosis in tissue between 25% and 50%	3
	Reversible damage with necrosis in tissue more than 50%	4
Interstitial	No damage	0
	Inflammation or haemorrhage exists	1
	Inflammation or haemorrhage exists with necrosis in tissue less than 25%	2
	Inflammation or haemorrhage exists with necrosis in tissue between 25% and 60%	3
	Inflammation or haemorrhage exists with necrosis in tissue more than 60%	4
Congestion	No congestion	0
	Congestion in tissue less than 25%	1
	Congestion in tissue between 25% and 50%	2
	Congestion in tissue between 51% and 75%	3
	Congestion in tissue between 76% and 100%	4

### Liver histopathological analysis

The liver was washed with physiological saline (0.9% NaCl) and immersed in a fixative solution (10% NBF). Histological preparation of the liver was carried out using the paraffin method, then stained with haematoxylin and eosin following Bancroft and Cook ^23^ with the same steps as kidney preparation. Histopathological scoring was performed using the ordinal post-examination masking method. Scoring was carried out in 100 fields of view per group at 40× objective lens magnification. Three parameters of damage, namely cellular damage, haemorrhage, and congestion were determined for the histopathologic scoring system ^26^ ^–^ ^28^ ( Table 2). Liver sample fixation and histopathological analysis were performed by the same researcher (SEDN).

**Table 2.** Histopathological scoring system for the liver.

Tissue type	Injury	Score
Cellular damage	No damage	0
	Reversible damage with necrosis in tissue less than 15%	1
	Reversible damage with necrosis in tissue between 15% and 40%	2
	Reversible damage with necrosis in tissue between 41% and 70%	3
	Reversible damage with necrosis in tissue between 71% and 100%	4
Haemorrhagic	No damage	0
	<15%	1
	15–40%	2
	41–70%	3
	71–100%	4
Congestion	No congestion	0
	Congestion in tissue less than 15%	1
	Congestion in tissue between 15% and 40%	2
	Congestion in tissue between 41% and 70%	3
	Congestion in tissue between 71% and 100%	4

### Data analysis

All measured data were analysed using appropriate methods and without any exclusions. Data were analysed qualitatively and quantitatively. Qualitative data analysis was carried out descriptively. For quantitative data analysis, a normality test was first carried out using the Shapiro-Wilk test (α=0.05). The scoring results were then statistically analysed to determine significant differences among groups (p<0.05) using the Kruskal-Wallis test. The test was continued with the Mann-Whitney test (α=0.05) since the data were not normally distributed. We used the Kruskal-Wallis test followed by the Mann-Whitney test to evaluate the effects of electric field exposure on structural damage to the kidneys and livers of healthy animals and tumour-bearing animals. Exposure to electric fields was the factor that determines structural damage to the kidneys and liver and was used as the basis for determining the single-factorial statistical tests used in this study. Different ways of comparing groups using the same statistical test will give different results, as seen in our second and third revisions of the article. All data were statistically analysed using SPSS program version 16 (RRID:SCR_002865) by the same researcher (NF).

## Results

The results of this study are a comparison of the histological characteristics of the kidney and liver under exposure to non-contact electric fields, which will be coherently described in the sections below.

### Histopathology of kidney

**Figure 2.**
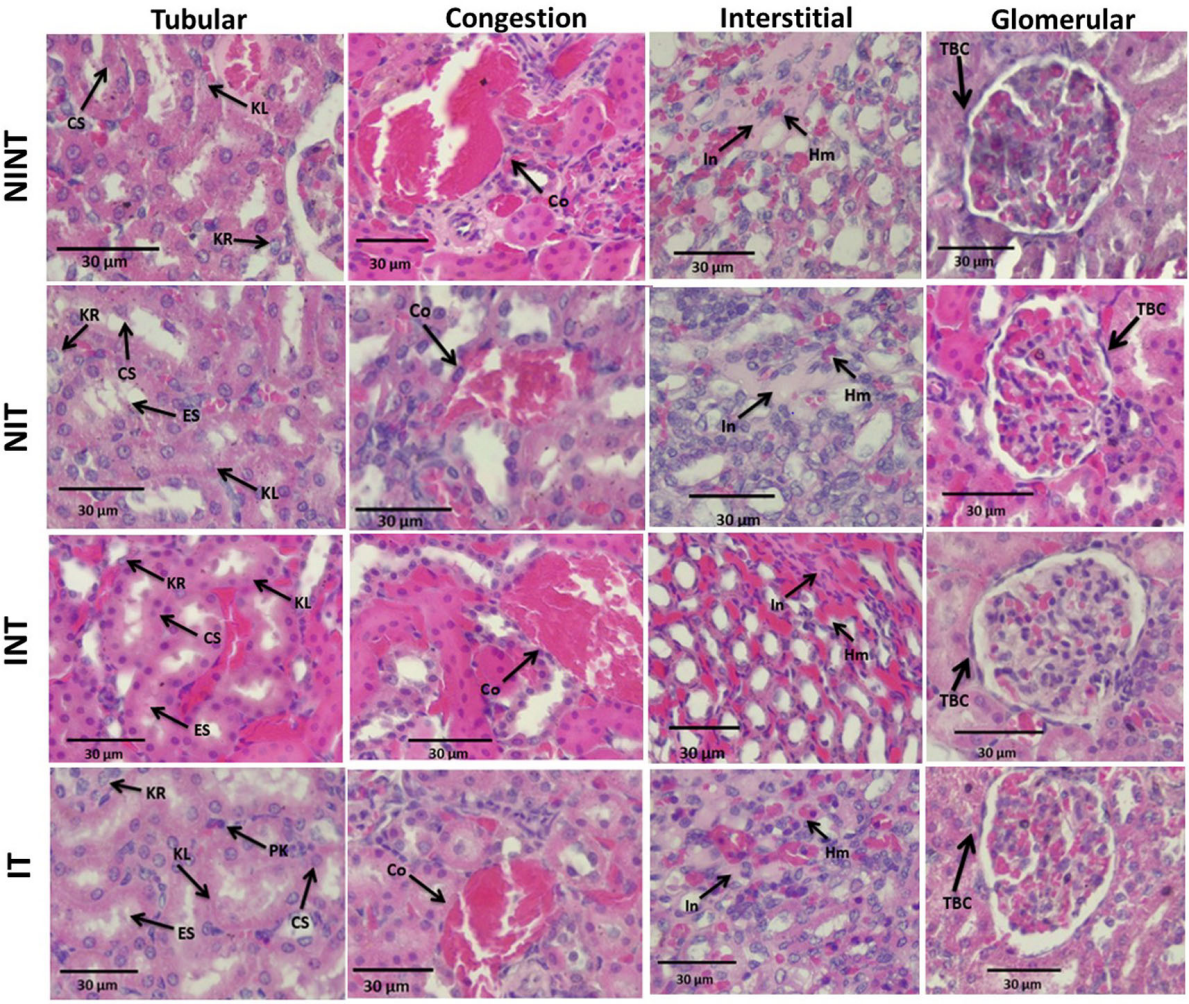
Histological features of tubular, interstitial, glomerular damages, and congestion in rat kidney sections stained with H&E. KL=Karyolysis, KR=karyorrhexis, PK=pyknosis, CS=cloudy swelling, ES=epithelial sloughing, Co=congestion, In=inflammation, Hm=haemorrhage, TBC=thickening of Bowman’s capsule, NINT=non-induction and non-therapy group, NIT=non-induction and therapy group, INT=induction and non-therapy group, and IT=induction and therapy group.

**Figure 3.**
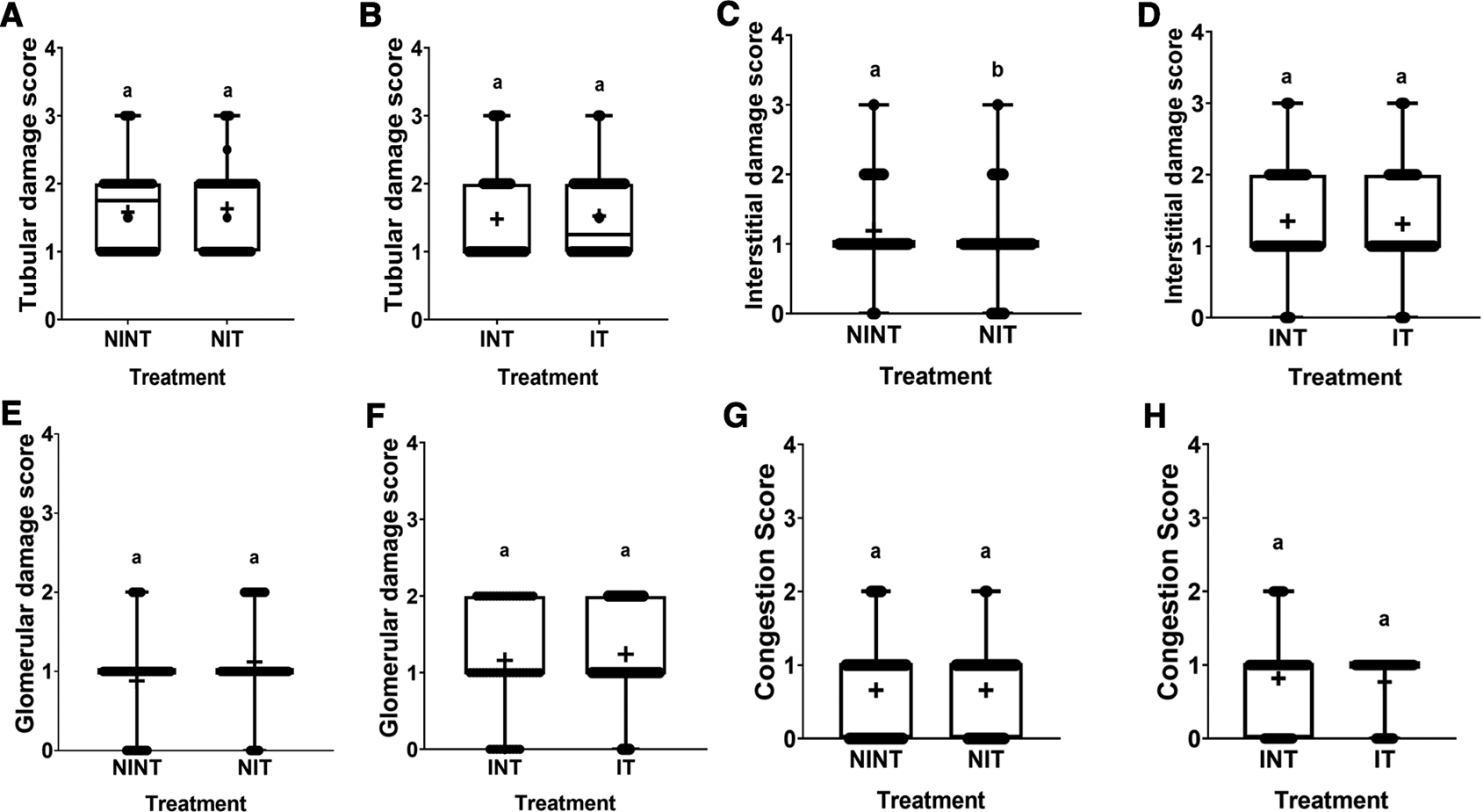
Scoring of tubular, interstitial, glomerular damages, and congestion in rat kidney sections. (A and B) Tubular damage, (C and D) interstitial damage, (E and F) glomerular damage, and (G and H) number of congestions.

The effects of non-contact electric field exposure on renal histopathology and renal damage scoring results are illustrated in Figure 2 and Figure 3, respectively. In addition, the results of histopathological scoring for the different groups for each parameter studied along with the p-value of differences between groups are presented in Table 3. Some damages were found in the renal tubules, including karyolysis, karyorrhexis, pyknosis, cloudy swelling, and epithelial sloughing. However, the damage scores were not significantly different (p>0.05), either in the kidneys of healthy rats ( Figure 3A) or in the kidneys of tumour-bearing rats ( Figure 3B). In the renal interstitial tissues, inflammation and haemorrhage were identified. Figure 3C shows the score of both damages in the NIT group (1.0±0.55) was significantly lower (p<0.05) than that in the NINT group (1.19±0.51). The main damage found in the renal glomerulus was thickening of the Bowman’s capsule. However, non-contact electric field exposure did not cause significant glomerular damage (p>0.05) in the kidneys of healthy rats ( Figure 3E) and the kidneys of tumour-bearing rats ( Figure 3F). Congestion was found as a common injury in all parts of the kidney structure. Similar to tubular and glomerular damages, the number of congestion was not significantly different (p>0.05), either in the kidneys of healthy rats ( Figure 3G) or in the kidneys of tumour-bearing rats ( Figure 3H).

**Table 3.** The kidney histopathological scoring results with p-values of difference between groups.

	NINT group	NIT group	INT group	IT group
Tubular damage score	1.58 ± 0.62	1.63 ± 0.60	1.48 ± 0.66	1.52 ± 0.56
	p = 0.5006		p = 0.2981	
Interstitial damage score	1.19 ± 0.51	1.00 ± 0.55	1.35 ± 0.63	1.31 ± 0.63
	p = 0.0108		p = 0.5618	
Glomerular damage score	0.88 ± 0.56	1.12 ± 0.56	1.16 ± 0.74	1.24 ± 0.59
	p = 0.0513		p = 0.6635	
Congestion score	0.66 ± 0.64	0.66 ± 0.54	0.82 ± 0.61	0.77 ± 0.42
	p = 0.7681		p = 0.7243	

### Histopathology of liver

**Figure 4.**
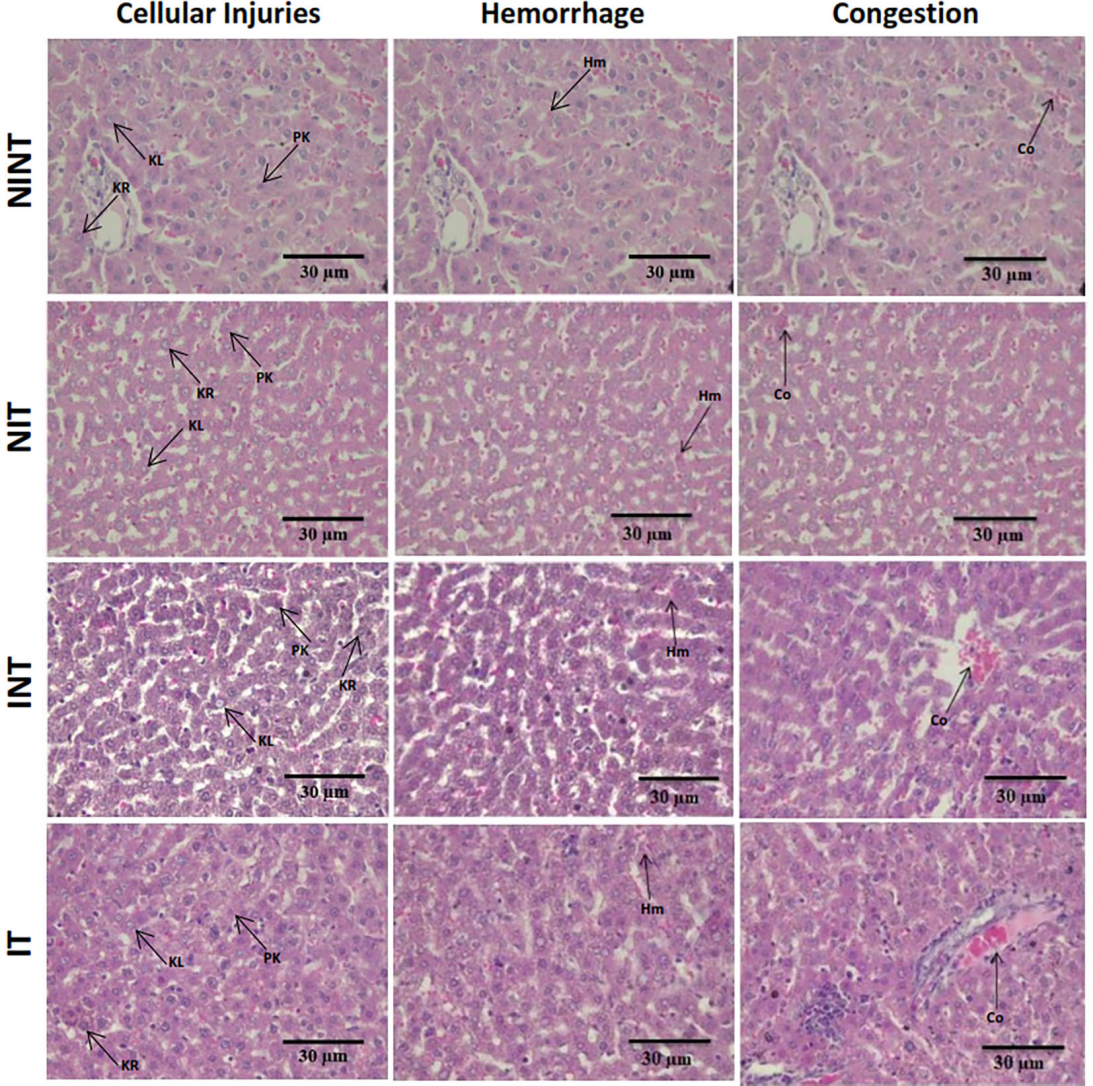
Histological features of haemorrhage, congestion, and cellular damage in rat liver sections stained with H&E. Hr=Haemorrhage, Cg=congestion, Pn=pyknosis, Kr=karyorrhexis, Kl=karyolysis, Cs=cell swelling, Fc=fatty change, NINT=non-induction and non-therapy group, NIT=non-induction and therapy group, INT=induction and non-therapy group, and IT=induction and therapy group.

**Figure 5.**
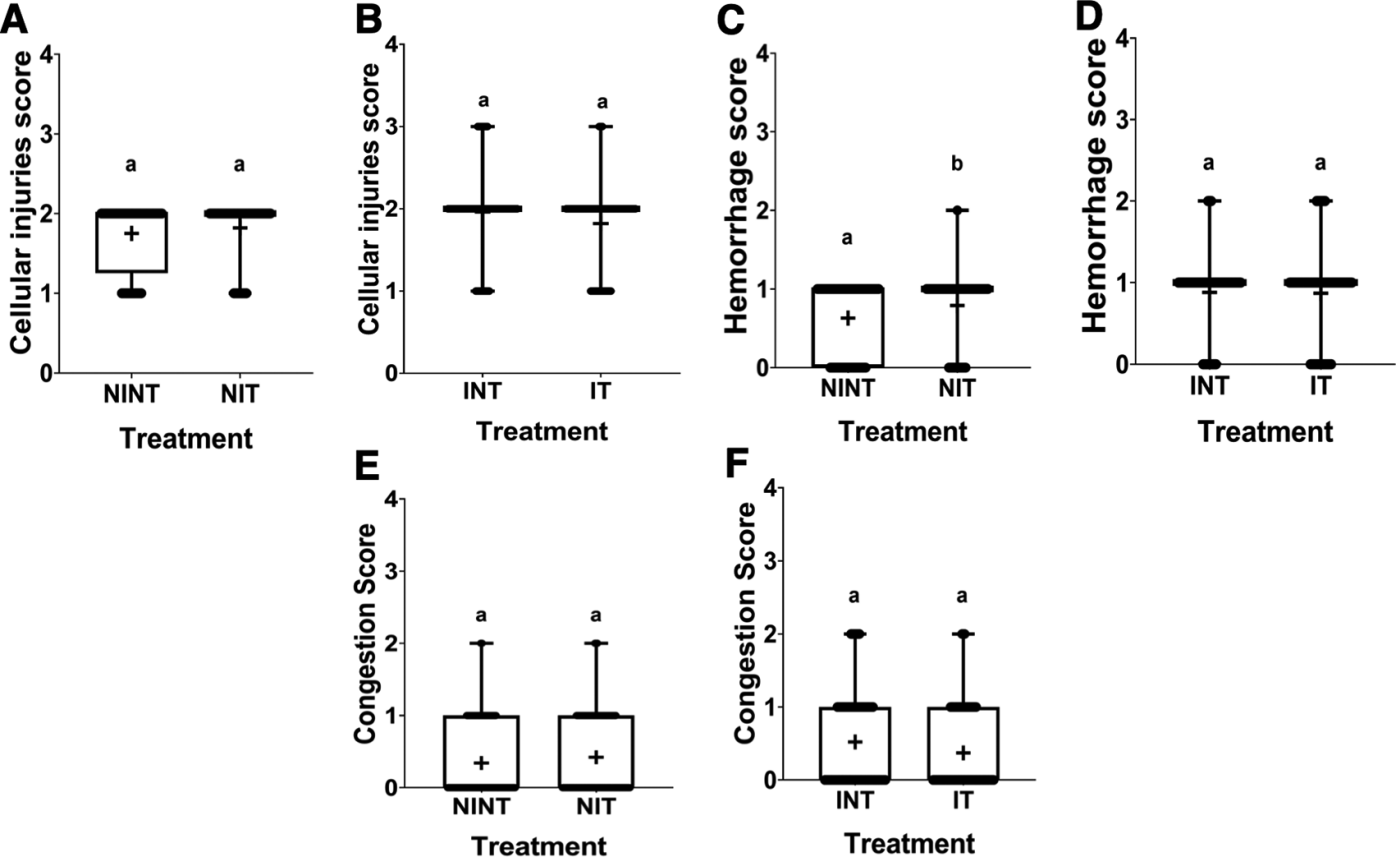
Scoring of cellular damage, haemorrhage, and congestion in rat liver sections. (A and B) Cellular damage, (C and D) haemorrhage, and (E and F) number of congestions.

The histopathological structure of the liver in the four groups had the same pattern of damage but with different levels of damage as shown in Figure 4 and Figure 5. In addition, the results of histopathological scoring for the different groups for each parameter studied are presented in Table 4. All groups experienced the same types of damage, namely cellular damage (pyknosis, karyolysis, karyorrhexis), haemorrhage, congestion, and reversible damage (cellular swelling and fatty changes). No significant cellular damage was found in the liver after exposure to non-contact intermediate-frequency electric fields (p>0.05), either in the livers of healthy rats ( Figure 5A) or in the livers of tumour-bearing rats ( Figure 5B). Figure 5C shows a significant difference in haemorrhage scores (p<0.05) in healthy rat livers between the NIT group (0.79±0.43) and the NINT group (0.63±0.48). The higher haemorrhage scores in the NIT group may indicate that the actively dividing liver cells were also sensitive to intermediate-frequency electric fields. However, there was no significant difference in haemorrhage scores (p>0.05) in the livers of tumour-bearing rats between the IT and INT groups ( Figure 5D). The scores of congestion were also not significantly different, either in the livers of healthy rats ( Figure 5E) or in the livers of tumour-bearing rats ( Figure 5F). Chronic hepatic congestion can eventually lead to hepatic fibrosis. The liver with hepatic congestion is histologically characterized by sinusoidal swelling and hemorrhagic necrosis in the perivenular area of the hepatic acini, leading to sinusoidal fibrosis and eventually forming fibrosis between adjacent central veins. ^29^ Liver fibrosis itself is characterized by excessive accumulation of extracellular matrix (ECM) proteins, ranging from mild pericellular fibrosis in the early stages to cirrhosis in the advanced stages, which is the common end stage of any liver disease. ^30^ Histology of the liver tissue in all groups did not show any fibrosis, so it can be said that the congestion that occurred was not yet at a chronic level. Since there was no significant difference in the scores of congestion and no fibrosis was found, congestion in all groups was still considered normal.

**Table 4.** The liver histopathological scoring results with p-values of difference between groups.

	NINT group	NIT group	INT group	IT group
Cellular damage score	1.75 ± 0.44	1.82 ± 0.39	1.96 ± 0.51	1.82 ± 0.48
	p = 0.3017		p = 0.0608	
Haemorrhage score	0.63 ± 0.49	0.79 ± 0.43	0.88 ± 0.46	0.87 ± 0.56
	p = 0.0220		p = 0.7963	
Congestion score	0.34 ± 0.52	0.42 ± 0.55	0.52 ± 0.66	0.37 ± 0.56
	p = 0.3180		p = 0.1147	

## Discussion

In the present study, the effects of non-contact intermediate-frequency electric fields were revealed in the results of histopathological analysis of the kidneys and livers in mammary tumour-bearing rats, as discussed below.

Thickening of the Bowman’s capsule as the main glomerular damage ( Figure 2) may be a result of glomerular hyperfiltration, ^31^ DMBA-induced nephrotoxicity, ^32^ and electric fields exposure. ^19^ Since no significant glomerular damage was observed in healthy rats (NIT group) and -tumour-bearing rats (IT group), non-contact electric field exposure did not affect the thickening of the Bowman’s capsule. Therefore, the electric field exposure may not alter the transmembrane potential and distribution of ion channels and dipoles. ^33^ Similar results were also shown in our other study using different electric field frequency (150 kHz) but the same intensity (50-60 V/m). In this study, thickening of the Bowman’s capsule was also found, but exposure to this electric field did not significantly affect the damage. ^19^

The nephrotoxic effects of DMBA occur not only in the glomerulus but also in the tubules. In addition, DMBA caused substantive nephrotoxicity characterized by renal tubular necrosis including karyolysis, karyorrhexis, and pyknosis, ^34^ as shown in Figure 2. Moreover, DMBA created obvious reversible histological changes in the tubules, such as epithelial sloughing and cloudy swelling, as illustrated in Figure 2. Epithelial sloughing represented progressive tubular disintegration, ^35^ and cloudy swelling may lead to cell necrosis. ^4^ Conversely, electric field exposure may direct the migration of mesenchymal stem cells to ameliorate acute nephrotoxicity ^36^ caused by DMBA. Therefore, electric field exposure did not increase renal tubular damage in tumour-bearing rats (IT group) as the difference in damage was not significant compared to untreated rats (INT group). In addition, non-contact electric field exposure also did not cause significant renal tubular damage in healthy rats (NIT group), so this electric field exposure does not harm the renal tubules. In our other study using different electric field frequency (150 kHz) but the same intensity (50-60 V/m), DMBA induction significantly damaged renal tubules. This suggests the nephrotoxic effect of DMBA on the renal tubules. In contrast, exposure to this electric field frequency resulted in decreased renal tubular damage in healthy rats (NIT group). Moreover, the frequency of this electric field may be able to compensate for tissue repair from damage caused by DMBA. ^19^ Therefore, exposure to intermediate-frequency and low-intensity non-contact electric fields was not harmful to the renal tubules. It can even repair the condition of damaged renal tubules.

In the renal interstitial tissue, the nephrotoxic effects of DMBA caused inflammation and haemorrhage, as shown in Figure 2. This inflammation can be affected by oxidative stress and can lead to impaired kidney function, including endothelial dysfunction, atherosclerosis, and glomerular injury. ^37^ Oxidative stress activates transcription factors including NF-kB, which activates the expression of inflammatory response genes. ^38^ In addition, Kandeel *et al.* ^39^ reported that oxidative stress may alter kidney structure and function due to the effects of reactive oxygen species (ROS) on mesangial and endothelial cells. Oxidative injury happens when ROS, including O _2_, H _2_O _2_, and -OH, ruin the cell’s antioxidant defense system. ^40^ These ROS can be produced due to DMBA administration ^41^ and can spread from their site of production to other sites inside the cell or even prolong the injury outside the cell. ^42^ Moreover, de Oliveira *et al.* ^43^ revealed that administering DMBA to develop tumours in animal models also caused haemorrhage. In contrast, exposure to this non-contact electric field significantly decreased the number of inflammation and haemorrhage in healthy rats (NIT group), as shown in Figure 3C and Table 3. Reducing inflammations and haemorrhages in the kidneys can reduce the risk of impaired kidney function. ^37^ In addition, reducing inflammation and haemorrhage in the kidneys can help protect the kidneys from damage, improve kidney function by improving the kidneys' ability to filter waste, reduce proteinuria, and reduce the risk of acute and chronic kidney diseases. ^44^ In other studies, exposure to high-frequency non-contact electric fields can also reduce inflammation, thereby accelerating wound healing in animal models. ^17^ To the best of our knowledge, these results are the first findings showing that non-contact electric field exposure can reduce inflammation and haemorrhage in rat kidneys. In our other study using a different electric field frequency (150 kHz) but the same intensity (50-60 V/m), renal interstitial injury was not significantly caused by exposure to this electric field. ^19^ In another study using electromagnetic field exposure with a frequency of 150 kHz in healthy rats, it showed normal kidney morphology, including normal-appearing glomeruli, tubules, and interstitium. ^45^ Therefore, exposure to intermediate-frequency electric fields was not harmful to renal interstitial tissue.

In contrast to kidney histology, there was significant damage, namely haemorrhage, in the liver of healthy rats (NIT group) after exposure to intermediate-frequency non-contact electric fields ( Figure 5C). Meanwhile, in tumour-bearing rats (IT group), no significant haemorrhagic damage occurred ( Figure 5D). Liver cells are actively dividing cells and have the same membrane potential as breast cancer cells. ^13^ ^,^ ^14^ With these characteristics, liver cells can be sensitive to exposure to electric fields. However, haemorrhage in the hepatic tissue does not show symptoms of acute haemorrhage, such as cellular hypoxia, decreased tissue perfusion, organ damage, and death. ^46^ In addition, different results were obtained in our other study using an electric field frequency of 150 kHz. Exposure to this electric field can significantly reduce haemorrhage in the liver of healthy rats (NIT group) and tumour-bearing rats (IT group). ^19^ In another study with magnetic field exposure, a frequency of 100 kHz could be tolerated by liver cells, so that exposure to this magnetic field did not affect the viability of normal liver cells. ^15^ In another study using exposure to a 150 kHz electromagnetic field, mild inflammatory changes with lymphocyte infiltration and haemorrhage were shown in the livers of healthy rats. This suggested possible liver damage or infection. However, the liver damage that occurred was insufficient to cause clinical and functional manifestations because the lesions were quite mild without significant changes in liver enzyme levels. ^45^ Based on the effects of 100 kHz electric field exposure on the livers of healthy animals, the use of this electric field frequency is only intended for cancer patients, not for healthy people or non-cancer patients.

The results in the therapy (IT) group with a lower hepatocellular damage score compared to the non-therapy (INT) group suggested that exposure to non-contact electric fields was not harmful to the livers of tumour-bearing rats, and even tended to repair hepatocellular damage. In addition, since the vascular congestion score was still within normal conditions and not at a chronic level, exposure to non-contact electric fields was not harmful. In our other study using an electric field frequency of 150 kHz with the same intensity (50-60 V/m), hepatocellular damage and congestion were not significantly induced by exposure to electric fields. ^19^ Therefore, exposure to intermediate-frequency electric fields was not harmful to the livers of the animals.

Damage to the kidneys and liver in healthy rats in the control group (NINT) could not be predicted because rats with disease symptoms were excluded. In addition, rats were also randomly selected for each group. Thickening of the Bowman’s capsule in the NINT group may occur naturally due to aging or ischemia. ^19^ ^,^ ^47^ Injury to the normal renal tubules may occur due to the high rate of reabsorption by the renal tubules. ^19^ For damage to the renal interstitial tissue, a score below 2 indicated that there was little inflammation or haemorrhage. Inflammation is part of the activation of the immune system in response to acute or chronic kidney injury which may be caused by pathogens that enter the rat’s body. ^48^ For damage to the liver in the NINT group basen on haemorrhagic and congestion scores with values below 1, indicating that the liver damage that occurred was very minor or nonexistent. For cellular damage scores below 2, this indicated that there was reversible damage with necrosis of less than 15%. Liver hepatocytes have many vital functions, so they can proliferate extensively, which allows efficient liver regeneration for reversible damage. ^49^ In addition, the liver itself is a very vulnerable organ due to its size and is the organ most frequently injured after abdominal trauma. ^50^

For the results of this study, we only report the effects of intermediate-frequency non-contact electric fields on the histological structure of the kidney and liver, not on their function. Kidney function parameters such as creatinine and bilirubin, and liver function parameters such as aspartate aminotransferase (AST) and alanine transaminase (ALT) will be reported along with the haematological profile of the rat blood. Based on the evidence of the efficacy and effects of ECCT on normal tissues and organs, ^8^ ^,^ ^9^ ^,^ ^19^ including the kidney and liver as reported in this study, we will conduct a phase I clinical trial of ECCT. The clinical trial for healthy volunteers will use the intermediate-frequency (100 kHz) electric field as used in this study.

## Conclusions

Exposure to non-contact electric fields with intermediate-frequency had various effects on kidney and liver tissues. Exposure to this electric field may cause haemorrhagic damage to the liver of healthy rats. However, in other liver tissues as well as the kidneys, exposure to this electric field was tolerable. In addition, exposure to this electric field did not cause significant haemorrhagic damage in tumour-bearing rats and could even reduce the number of inflammations and haemorrhages in the kidneys of healthy rats.

## Ethical approval

This research was carried out at the LPPT UGM and the Animal Structure and Development Laboratory of the Faculty of Biology, UGM. LPPT UGM has been awarded ISO/IEC 17025:2000 accreditation for competence in testing and calibration. ^11^ Experimental protocol in this research was performed following approval by the Ethical Clearance Committee of LPPT UGM with ethical clearance number: 00015/4/LPPT/IV/2017, that has been previously reported. ^9^ The Ethical Clearance Committee stated that this research met the ethical requirements for the study on experimental animals and that the Ethical Clearance Committee had the right to conduct monitoring during the research.

## Data Availability

Open Science Framework: Kidney and liver histology in tumour-induced rats exposed to non-contact electric fields, https://doi.org/10.17605/OSF.IO/54BYF. ^51^ This project contains the following underlying data:
• Kidney and liver histological images • Kidney scoring and statistical analysis • Liver scoring and statistical analysis • Kidney and liver charts Open Science Framework: Kidney and liver histology in tumour-induced rats exposed to non-contact electric fields, https://doi.org/10.17605/OSF.IO/54BYF. ^51^ This project contains the following extended data:
• Ethical clearance document Open Science Framework: ARRIVE checklist for ‘Kidney and liver histology in tumour-induced rats exposed to non-contact electric fields’, https://doi.org/10.17605/OSF.IO/54BYF. ^51^ Data are available under the terms of the Creative Commons Zero “No rights reserved” data waiver (CC0 1.0 Public domain dedication).
