# Supplementary material for: Effects of non-contact electric fields on the kidneys and livers of tumour-bearing rats
Source: F1000Res. 2025 Feb 11;12:117. Originally published 2023 Feb 1. [Version 6] doi: 10.12688/f1000research.110080.6 (PMC11809679; doi:10.12688/f1000research.110080.6)
Supplement: Peer review history

# Reviewer report: Reviewer response for version 6

**DOI:** 10.5256/f1000research.177801.r365756

**Published:** 2025-02-19

**Authors:** Mohamed Hisham Fouad Aref

**Affiliations:** 1 Biomedical Engineering Researcher, Cairo, Egypt

Dear Editor and Authors,

 I really appreciate all the hard work done by the Authors to edit and enhance their article,

 looking forward to see their work soon.

 Best regards.

Is the work clearly and accurately presented and does it cite the current literature?

Partly

If applicable, is the statistical analysis and its interpretation appropriate?

Partly

Are all the source data underlying the results available to ensure full reproducibility?

Partly

Is the study design appropriate and is the work technically sound?

Yes

Are the conclusions drawn adequately supported by the results?

Yes

Are sufficient details of methods and analysis provided to allow replication by others?

Partly

Reviewer Expertise:

Biomedical Engineering, Hyperbaric Oxygenation, Biomedical Enhancement, Biomedical Applications, Hyperspectral Image Analysis

I confirm that I have read this submission and believe that I have an appropriate level of expertise to confirm that it is of an acceptable scientific standard.

# Reviewer report: Reviewer response for version 5

**DOI:** 10.5256/f1000research.174805.r349306

**Published:** 2024-12-31

**Authors:** Mohamed Hisham Fouad Aref

**Affiliations:** 1 Biomedical Engineering Researcher, Cairo, Egypt

The article is presenting one of the interesting subjects related to kidney and liver tumors. Additionally, the authors did a great job to enhance their previous versions. However, I have thoroughly reviewed the article, the article still needs some additional editing and enhancement to be ready for next step and indexing. The following aspects of the paper should be addressed for improvement:

1. The current title is specific, clearly indicating the study's focus on the effects of non-contact electric fields on kidney and liver histology in tumor-induced rats. However, It would benefit from revision by the authors to make it shorter, more concise, and informative, capturing the reader’s attention with clear and appealing language.
2. The authors are preferred to add “highlights bullets” to elaborate the importance of their research and grab the reader`s attention.
3. The “Abstract” section it needs to be revised by the authors and re-editing in a better presentable manner without skipping the ideas to comply with the presented idea of the manuscript and more organized.
4. Kindly in the “ **Abstract**” section have several week points which needs to be enhanced, such as:

- It lacks detail on the sample size and the extent of variability in observed results.
- Phrases like “safe in some contexts” are vague and could benefit from precise language to avoid ambiguity.

1. The “ **Introduction**” section thorough background on the potential of non-contact electric field therapy (ECCT) and its relevance in cancer treatment. However, it still needs to be revised and enhanced in each of the following:

- Repetition of points about electric field effects (e.g., intermediate-frequency benefits) could have been condensed.
- Does not clearly state the specific hypothesis or expected outcomes of the study.
- Lacks detailed justification for the chosen frequency (100 kHz) compared to previously studied 150 kHz.

1. Kindly read some of these useful previous researches with different and various techniques toward your study (Tumor detection) which could be helpful in your “Introduction and Literature sections” for your research outcome and could be more valuable to your research, feel free to **use** **them in your study or** **not** after reading them:

- Zhang X, et al., 2024 (Ref 1)
- Matos LC, et al., 2021 (Ref 2)
- * *Aref MH, et al., 2024 (Ref 3)
- *Sequin, Emily Katherine. Effects of induced electric fields on tissues and cells. Diss. The Ohio State University, 2014, https://etd.ohiolink.edu/acprod/odb_etd/etd/r/1501/10?clear=10&p10_accession_num=osu1403869854.*
- *Rijal, Nava P. Wireless non-contact electric field therapy improves healing in a porcine model. Diss. University of Cincinnati, 2021 https://www.proquest.com/openview/a8b24b1ee8cb580713289e3734531643/1?pq-origsite=gscholar&cbl=18750&diss=y.*

 

1. The “ **Materials & Methods **section is detailed, with clear descriptions of experimental design, animal care, and histological procedures. On the other hand, the section needs to be revised and re-edit regarding the following recommendations:

- Limited sample size (2 kidneys and livers per group) raises concerns about the statistical power and generalizability of results.
- It is unclear why data from other animals in each group were not included, which undermines transparency.
- Does not provide justification for the specific exposure duration (10 hours daily for 21 days) or how this relates to potential clinical applications.

 

1. The “ **Result**” section is presented with statistical analysis, including p-values for differences between groups. However, it needs the following:

- Key findings, such as the reduction of kidney inflammation and liver hemorrhage, are not adequately contextualized with broader implications.
- Some results, such as the lack of fibrosis or chronic congestion, are mentioned but not sufficiently explained.
- Limited discussion of variability in results across groups, which could indicate inconsistencies.

 

1. The “ **Discussion**” section provides a logical interpretation of findings, linking observed changes to potential mechanisms (e.g., oxidative stress, immune responses). For improvement, kindly see the following recommendations:

- Overemphasis on certain findings (e.g., kidney safety) while underexploring significant liver damage in healthy rats.
- Speculative claims, such as "this therapy may be used to treat kidney injuries," lack experimental validation.
- The discussion of ECCT parameters (frequency, intensity) is insufficient; no clear rationale is given for choosing these specific conditions.
- Does not address the broader implications of findings for clinical translation, such as potential risks in non-cancer patients.
- Combine previous researches toward your study and compared it with your findings, feel free to use other techniques to compare your study`s technique.

 

1. Kindly revise the “ **Reference”** section and update it, to cover your study`s background and literature review. Additionally, to be in the latest and nearest year to your research, as some reference needs to be replaced with the updated researches, such as: (Ref #1-2005 / Ref #2-2010 / Ref #3-2007 / Ref #6-2003/ Ref #18-2010 / Ref #19-2003…etc.).
2. The English language in this manuscript requires considerable improvement, as it contains several typographical errors and overly long sentences with mixed ideas, which reduce clarity and impact. I recommend the authors revise the manuscript with more concise and well-structured sentences.

In conclusion, I commend the authors for their hard work and efforts in preparing this manuscript. However, I believe further refinement is needed to improve the organization of ideas and to more clearly present the study's findings with additional detail and clearer illustrations.

**Best regards and good luck,**

Is the work clearly and accurately presented and does it cite the current literature?

Partly

If applicable, is the statistical analysis and its interpretation appropriate?

Partly

Are all the source data underlying the results available to ensure full reproducibility?

Partly

Is the study design appropriate and is the work technically sound?

Yes

Are the conclusions drawn adequately supported by the results?

Yes

Are sufficient details of methods and analysis provided to allow replication by others?

Partly

Reviewer Expertise:

Biomedical EngineeringHyperbaric OxygenationBiomedical EnhancementBiomedical ApplicationsHyperspectral Image Analysis

I confirm that I have read this submission and believe that I have an appropriate level of expertise to confirm that it is of an acceptable scientific standard, however I have significant reservations, as outlined above.

## References

[1] : A new tumor-treating device OM-100 with low-frequency magnetic fields inhibits proliferation and metastasis in liver cancer. *BMC Cancer* .2024;24(1) : 10.1186/s12885-024-13121-9 1383 10.1186/s12885-024-13121-9 39528972 PMC11552396

[2] : Perspectives, Measurability and Effects of Non-Contact Biofield-Based Practices: A Narrative Review of Quantitative Research. *Int J Environ Res Public Health* .2021;18(12) : 10.3390/ijerph18126397 10.3390/ijerph18126397 34199174 PMC8296239

[3] : Custom Hyperspectral Imaging System Reveals Unique Spectral Signatures of Heart, Kidney, and Liver Tissues. *Spectrochim Acta A Mol Biomol Spectrosc* .2024;305: 10.1016/j.saa.2023.123363 123363 10.1016/j.saa.2023.123363 37776837

# Response

**Published:** 2025-02-01

**Authors:** Firman Alamsyah

**Affiliations:** Ctech Labs Edwar Technology, Indonesia

We would like to thank the reviewer who have read our manuscript in detail and provided valuable input, as well as providing appropriate reference recommendations. We have made improvements to our manuscript based on the reviewers' suggestions and responded to all of the reviewers' notes, as we have written below.

 1. We have revised the title to be shorter, more concise and informative, in order to attract readers' attention with clear and engaging language.

 2. We do not know where to add “highlights bullets” and whether this is allowed or not by the journal editor. The importance of our research has been explained in the Introduction.

 3. We have revised and re-edited the Abstract without eliminating any ideas to make it more consistent with the ideas presented in the manuscript and to make it more organized.

 4. We have added details regarding sample size and the level of variability in observed results in the Abstract. We have replaced the word "safe" with another word that is more appropriate according to the context of the sentence.

 5. We have omitted the repetition of points about the effects of electric fields to make it more concise. We have refined the hypothesis to be clearer and more specific. We have added detailed justification for the 100 kHz frequency compared to 150 kHz.

 6. We have read the recommended studies provided by the reviewer and have compared them with the techniques and results of our study.

 7. We only took 4 organs from 2 individual animals, and did not take organs from other individuals, because we considered the number of samples used for histological examination to be representative enough and had been approved by the ethics committee. We have added justification regarding the exposure duration of 10 hours per day for 21 days.

 8. We have adequately contextualized the reduction in renal inflammation and hemorrhage with broader implications. We have adequately explained the relationship between fibrosis and chronic congestion. Based on suggestions from previous reviewers, we compared between 2 groups only, namely the effect of electric field on healthy rats (NIT vs NINT) and the effect of electric field on tumor-bearing rats (IT vs INT).

 9. We have removed the term kidney safety and the sentence: "this therapy may be used to treat kidney injuries". We have added a clear rationale for using the frequency (100 kHz) and intensity (59-60 V/m) of ECCT in the Introduction section. We do not discuss these ECCT parameters in the Discussion section because these parameters are not the results of this study. We have added implications of using 100 kHz electric fields in non-cancer patients. We have combined several previous studies provided by the reviewer and compared them with our research techniques and results.

 10. We have replaced all references published before 2011 with references published within the last 9 years. We also added several new references published in the last 10 years, including references recommended by the reviewer.

 11. We've fixed typographical errors and broken up the overly long sentences into two shorter sentences.

# Reviewer report: Reviewer response for version 4

**DOI:** 10.5256/f1000research.160919.r303396

**Published:** 2024-08-26

**Authors:** Tejaswi Worlikar

**Affiliations:** 1 University of Michigan, Ann Arbor, MI, USA

The authors present a follow-up in vivo study investigating the safety of non-contact electric fields on healthy liver and kidney in Sprague Dawley breast cancer rat model using different settings of ECCT therapy compared to their original study. The following suggestions are provided to improve the quality of this manuscript:

 1. Introduction:

 "In our preliminary study using 9 mice, the intermediate-frequency (100 kHz) electric fields of Electro-Capacitive Cancer Therapy (ECCT) gave good results, wherein the tumour size was reduced by more than 67%, but showed no histological alterations in mammary and skin tissues."

 Can you provide information on the effects of ECCT on tumor size in the current study? Did you observe tumor reduction? The previous published version of the study does not include any figures or tables showing the claimed 67% reduction in tumor size, can you please provide this as well?

 2. Necropsy and organ harvesting: "Two kidneys and two livers from different rats were randomly collected from each group. A total of 16 organs were used for histological examination." If each group had 6 rats, can you please elaborate why only two kidneys and two rats were collected from each group? Is there no data available on the remaining 4 rats? If so, then the actual sample size for your safety analysis becomes quite small at 2 samples/group and 4 groups total.

 3. Conclusions: "Exposure to this electric field can cause haemorrhagic damage in the livers of healthy rats, however, in other liver tissues and the kidneys, exposure to this electric field was safe. In addition, exposure to this electric field did not cause significant haemorrhagic damage in rats with breast cancer. It can even decrease the number of inflammations and haemorrhages in the kidneys." I am not sure if there is sufficient data presented to support the statement "can even decrease the number of inflammations and haemorrhages in the kidneys", since the sample size is low. A similar negative conclusion can be drawn saying "It can even increase the number of haemorrhages in the livers" (based on Table 4 data), but this would be inaccurate. Please consider rephrasing the conclusions. 

 4. Table 4 - Is this table showing the liver histopathological scoring results? Currently both Table 3 and Table 4 have the same heading. Please revise.

 5. What are the differences in ECCT therapy parameters between the current study and the previous study  "Nurhidayat L, Fajar I, Yati A, *et al. *2022 [Ref-1]"? The current study uses 100 kHz and the previous study used 150 kHz, and both used 18 Vpp, is this assessment correct? Can you provide some insights into why these parameters were chosen/modified? Please mention the other study in your introduction, especially when it is claimed that this may be the first study to  investigate the abnormalities in the kidney and liver under exposure to 100 kHz intermediate-frequency and low-intensity (50-60 V/m) non-contact electric fields.  

 6. "Damage to the kidneys and liver of the rats in the control group (NINT) cannot be predicted because rats with symptoms of illness had been excluded and rats were also randomly selected for each group." Were any rats excluded after the start of DMBA administration and/or ECCT therapy? If yes, what is the final size of each group? What types of illness were observed?

 7. "Moreover, since this electric field exposure can reduce the number of inflammations and haemorrhages in the kidneys, this therapy can be used to treat kidney injuries or related diseases." Please consider rephrasing this claim.

Is the work clearly and accurately presented and does it cite the current literature?

Partly

If applicable, is the statistical analysis and its interpretation appropriate?

Yes

Are all the source data underlying the results available to ensure full reproducibility?

Partly

Is the study design appropriate and is the work technically sound?

Partly

Are the conclusions drawn adequately supported by the results?

Partly

Are sufficient details of methods and analysis provided to allow replication by others?

Partly

Reviewer Expertise:

Small animal tumor models, cancer therapy, histotripsy

I confirm that I have read this submission and believe that I have an appropriate level of expertise to confirm that it is of an acceptable scientific standard, however I have significant reservations, as outlined above.

## References

[1] : Evaluation of Static Electric Field Exposure on Histopathological Structure and Function of Kidney and Liver in DMBA-Induced RAT (Rattus norvegicus Berkenhout, 1769). *Malaysian Journal of Fundamental and Applied Sciences* .2022;18(6) : 10.11113/mjfas.v18n6.2725 703-713 10.11113/mjfas.v18n6.2725

# Response

**Published:** 2024-11-13

**Authors:** Firman Alamsyah

**Affiliations:** Ctech Labs Edwar Technology, Indonesia

First of all we would like to apologize for the delay in responding to reviewer questions. We have answered the questions in the order given and have revised the article according to the reviewers' suggestions. We are very grateful to the reviewers who have evaluated this article and provided suggestions for improving this article. We hope that the reviewer will be willing to re-read the article that we have revised.

 1. We realize that we did not include the graph of tumor size reduction of more than 67% in the previous publication that we cited in this article. However, we have data on tumor size reduction after therapy in publication number 9 (Figure 2) in the reference list: Alamsyah F, Pratiwi R, Firdausi N, et al.: Cytotoxic T Cells Response with Decreased CD4/CD8 Ratio During Mammary Tumors Inhibition in Rats Induced by Non-Contact Electric Fields. *F1000 Res.* 2021;10(35):1–21.

 2. Yes, we only took samples of 2 kidneys and 2 livers from each group. We did not take kidneys and livers from all rats, so we do not have the data. The data of 2 kidneys and 2 livers from each group, we consider quite representative.

 3. We have rephrased the conclusion as follows “Exposure to this electric field may cause haemorrhagic damage in the livers of healthy rats, however, in other liver tissues and the kidneys, exposure to this electric field was safe. In addition, exposure to this electric field did not cause significant haemorrhagic damage in rats with breast cancer and it may decrease the number of inflammations and haemorrhages in the kidneys of healthy rats.”

 4. We have revised the heading of Table 4.

 5. Yes, it is correct. We used a 100 kHz frequency electric field based on the results of a pilot study using mice exposed to a 100 kHz frequency electric field. We have also written in the Introduction that the electric field of 100 kHz gave the best results in our cell studies. Then we used a frequency of 150 kHz in a different study to investigate the difference in electric field frequencies in cancer therapy and also its safety on normal tissues. We have mentioned other study using 150 kHz frequency electric fields in the Introduction.

 6. Rats were only excluded before and after the start of DMBA administration. We used 40 rats in total for this study and rats that had symptoms of illness were excluded before rats were assigned to each group. Rats that had symptoms of illness were rats that had lost weight. The final size of each group was 6 rats.

 7. We have rephrased the sentence as follows “Moreover, since this electric field exposure may reduce the number of inflammations and haemorrhages in the kidneys, this therapy may be used to treat kidney injuries or related diseases.”

# Reviewer report: Reviewer response for version 3

**DOI:** 10.5256/f1000research.157543.r218329

**Published:** 2023-11-22

**Authors:** Michael Staelens

**Affiliations:** 1 Instituto de Física Corpuscular (IFIC), Paterna, Valencia, Spain

Although certain aspects of the article are improved, notable issues persist (several of which have already been mentioned yet remain unresolved), casting uncertainty over the study’s conclusions and hindering overall comprehension. In particular, numerous inconsistencies, insufficiencies in the presentation of the results, and a general lack of clarity pose significant challenges in following its content and undermine confidence in its conclusions. These issues must be resolved to ensure that the study and its conclusions can be accurately understood and interpreted by the scientific community. Specific issues that must be rectified—several of which have already been stated clearly in past reports—and various suggestions to facilitate timely revisions are as follows:

1. There appear to be serious inconsistencies between the conclusions of the study stated in various sections of the article.
  The Abstract states, “ *The number of inflammation and hemorrhage in the kidney structure of the placebo group was the lowest and significantly different from the three other groups. All damages in the kidney were also found in the liver, but each showed different levels of damage. **The damages in the kidney and liver caused by the exposure were not significant.***”
  Then, in the Discussion section, the following is stated: “ *In contrast to kidney histology, **there was a significant damage, namely hemorrhage, in the liver of healthy rats (NIT group) after exposure to intermediate frequency non-contact electric fields***”.
  Further examples of these inconsistencies in the stated results/conclusions are present. This lack of alignment in reporting the findings of the study must be reconciled for clarity and to ensure an accurate interpretation of the study by the scientific community.
2. The presentation of results, in general, lacks clarity. It would be very helpful to the reader to include a table presenting all of the histopathological scoring results for the different groups (i.e., the mean ± SD for each of the groups for each of the parameters studied to quantify damage). This would enhance the clarity and transparency of the presented results and also allow readers to verify/reproduce the results of the statistical tests.
3. This is also the case regarding the results of the statistical hypothesis tests, in which the full results are not clearly presented in the manuscript. The inclusion of the results of these tests (possibly also in an additional table) would be beneficial in helping the reader to more easily identify and understand what differences were significant. Additionally, when significant differences, or the lack thereof, are presented, the *p*-value should be stated rather than simply *p*<0.05 or *p*>0.05; this is a better practice in scientific reporting and is important for the interpretation of the results so that the reader can understand not simply if a difference obtained in the study was significant or not, but also *how* significant (i.e., at what level of significance).
4. Many statements in the article regarding significant differences would benefit from stating the *p*-value for additional support and clarity around the significance of the observed difference. For example, “ *exposure to this non-contact electric field significantly decreased the number of inflammations and haemorrhages in the healthy rats (NIT group),*” and “ *DMBA induction significantly damaged renal tubules.*”
5. Some statements do not follow from the statistical test results reported and should be removed. For example, in the section on “ *Histopathology of liver*”, the authors report “ ***no significant difference in hemorrhage scores (p>0.05) in the livers of rats with breast cancer between the IT and the INT groups***”; however, the next sentence reads, “ *Exposure to intermediate frequency electric field in the IT group slightly **decreased hemorrhage in the liver compared to the INT group***”. This directly contradicts the previous statement that no significant difference was found. Moreover, comparing the values reported, it is obvious to the reader that there is absolutely no statistically meaningful difference (0.87±0.56 vs. 0.88±0.46), i.e., no decrease that can or should be reported. The full passage is the following: “ *However, there was no significant difference in hemorrhage scores (p>0.05) in the livers of rats with breast cancer between the IT and the INT groups (Figure 5D). Exposure to intermediate frequency electric field in the IT group slightly decreased hemorrhage, cellular injury and congestion in the liver (0.87±0.56, 1.82±0.48, 0.37±0.56, respectively) after DMBA administration compared to the INT group (0.88±0.46, 1.96±0.51, 0.52±0.66, respectively).*” Only the statistically meaningful differences should be reported as observed effects from the EF exposure and with the *p*-values explicitly stated.
6. Regarding the choice of statistical tests, the decision to analyze factors separately and exclusively conduct one-way tests is technically acceptable, albeit at the cost of sacrificing some information and statistical power. I suggest the authors consider one of the following options:
  - Since the authors’ response states that they were unsuccessful in applying the SRH extension of the Kruskal–Wallis test, an alternative method that is highly applicable and more suitable for their study design than repeated one-way tests is **ordinal logistic regression**, which can be thought of as a multifactorial generalization of the Kruskal–Wallis test. This would ultimately be preferable to the statistical analysis methodology currently employed in the most recent version of the article and would enhance the interpretation of the results. For guidance on applying ordinal logistic regression in SPSS, please refer to the following page: https://statistics.laerd.com/spss-tutorials/ordinal-regression-using-spss-statistics.php.
  - If the authors do not wish to attempt the previous suggestion or are unsuccessful in doing so, then the article should include at least some discussion on the rationale behind the chosen statistical testing methodology, as well as provide further insights into the drawbacks and caveats resulting from this choice.
7. The overall clarity and correctness of the writing still need work. Please perform a final series of revisions to the writing and unify English spellings. I strongly recommend using a free writing tool to help improve the writing (e.g., Grammarly), which would catch most of the remaining errors. A few examples:
  - “ *In another study using electromagnetic field exposure to 150 kHz in healthy SD rats, showed normal kidney morphology, including normal-appearing glomeruli, tubules, and interstitium*”. Revise to “Another study using electromagnetic field exposure at 150 kHz in healthy SD rats showed…”
  - “ *The effects of non-contact electric fields exposure*”. “ *electric fields exposure*” -> ”electric field exposure”.
  - “ *Fresh samples of the kidney and liver were collected for observing structural damage in both organs*.”   “ *for observing*” -> “to observe”.
  - Both “ *haemorrhage*” and “ *hemorrhage*” are written. Please maintain one choice of English spellings consistently and revise all instances of varied spellings to conform to this choice (preferably the former spelling, as the manuscript seems to largely conform to British English spellings).
  - Number ranges, e.g., “50-60 V/m”, should use an en dash rather than a hyphen, i.e., 50–60 V/m.

Is the work clearly and accurately presented and does it cite the current literature?

Partly

If applicable, is the statistical analysis and its interpretation appropriate?

No

Are all the source data underlying the results available to ensure full reproducibility?

Yes

Is the study design appropriate and is the work technically sound?

Partly

Are the conclusions drawn adequately supported by the results?

No

Are sufficient details of methods and analysis provided to allow replication by others?

Partly

Reviewer Expertise:

Electromagnetic interactions with biological systems; non-invasive therapies; microtubules; biophysics; high energy physics; particle physics.

I confirm that I have read this submission and believe that I have an appropriate level of expertise to state that I do not consider it to be of an acceptable scientific standard, for reasons outlined above.

# Response

**Published:** 2024-01-02

**Authors:** Firman Alamsyah

**Affiliations:** Ctech Labs Edwar Technology, Indonesia

Dear reviewer,

 I have revised the article and written my response according to your suggestions and requests.

 1. There is an error in the article update carried out by F1000 Research. Specifically for the abstract, F1000 Research did not update revisions to the abstract in our article. The abstract written in the 2nd revision of our article is the first abstract we wrote. In addition, F1000 Research did not ask us for confirmation first, before the second revision of our article was published. We have reported the issue to the journal editor.

 The original revision we wrote in the abstract was as follows: “All damages to the kidneys were also found in the liver, but each showed a different degree of damage. Exposure to this electric field can cause haemorrhagic damage to the livers of healthy rats, but not to rats with breast cancer.”. We have rewritten all revisions to the abstract.

 2. Kidney and liver scoring and statistical analysis can be accessed on the link in the Underlying data section. However, we have presented the data in a table in our latest article revision according to the reviewer’s request.

 3. Similar to response number 2, kidney and liver scoring and statistical analysis can be accessed on the link in the Underlying data section. However, we have added the p-values in our revised article according to the reviewer’s request in Table 3 and Table 4.

 4. We have added the p-values in our revised article in Table 3 and Table 4.

 5. We have removed the sentence that contradicts the statistical results.

 6. We maintain the statistical tests used in our article and have added several reasons for determining these statistical tests along with their weaknesses.

 7. We have used Grammarly to correct spelling mistakes in English in our revised article.

# Reviewer report: Reviewer response for version 2

**DOI:** 10.5256/f1000research.148608.r175322

**Published:** 2023-06-02

**Authors:** Michael Staelens

**Affiliations:** 1 Instituto de Física Corpuscular (IFIC), Paterna, Valencia, Spain

The revised version of the manuscript, “Kidney and liver histology in tumour-induced rats exposed to non-contact electric fields”, by Alamsyah *et al*., is an improvement over the first version, with many of the reviewers’ suggestions implemented. However, Major Concern #1 from my first review of the manuscript does not appear to have been taken into account; despite the authors’ claim that a new statistical analysis was performed, the Data Analysis section still states that “ *scoring results were then analysed statistically to determine significant differences among groups (p<0.05) using the **Kruskal-Wallis test followed by the Mann-Whitney test***”. As highlighted in my first review of the manuscript, this is an incorrect choice of statistical analysis methodology. This is problematic regarding both the interpretation of the data and the subsequent conclusions drawn from the study. Two alternative non-parametric methods (i.e., do not require normally distributed data) that can be used when a measure is affected by two (or more factors) and are thus applicable to the results reported by the authors in the manuscript were suggested; however, neither one appears to have been implemented in the revisions.

 The alternating “ *non-contact electric fields*” therapy studied by the authors is nearly identical to the relatively recently FDA-approved alternating electric field therapy—often referred to in the literature as tumor-treating fields—which (instead) typically uses electrodes placed on the skin and has shown remarkable efficacy as a non-invasive anticancer treatment modality. Consequently, the most important aspects of the study reported by the authors (as far as I can tell) are how the experimental group that received both tumor induction and EF therapy compares to the other experimental/treatment groups and the control group, as well as how the EF-only group and the control group compare (which is interesting for establishing effects of the treatment on healthy/non-tumoral cells). There are four groups reported in the manuscript, control (NINT), EF only (NIT), tumor induction only (INT), and the combined tumor induction and EF therapy group (IT). The groups thus **differ** very clearly **by two independent variables** (comprising two levels each): EF treatment (yes/no) and tumor induction (yes/no). The Kruskal–Wallis test **cannot** be used to compare these groups; the same is true of the Mann–Whitney test, which **assumes that the groups differ by only one independent variable**. To phrase it differently, the Kruskal–Wallis test is a **one-way test**; comparing the control to the IT group, for example, involves comparing groups that differ in **two ways**. A simultaneous comparison between the IT group and the three other groups must be performed in order to correctly establish the influence of the two different independent variables on the dependent variable measured (i.e., the histopathological scoring results) and to determine the main effect of each independent variable as well as to determine if there is any interaction between the two independent variables.

 “[The] *selection of [an] appropriate statistical method is [a] very important step in [the] analysis of biomedical data. A wrong selection of the statistical method not only creates some serious problem[s] during the interpretation of the findings but also affects the conclusion[(s)] of the study*” ^1^. Consequently, the key results and conclusions stated in the manuscript might be erroneous (i.e., false claims of significance/insignificance). Thus, the status of my review of the manuscript remains “not approved”. The problem is that without correctly applying an appropriate statistical analysis method that suits the data collected and the results obtained, the actual findings and conclusions of the study are unclear. Therefore, until this major concern is resolved, I do not believe that the manuscript and the reported results should be indexed or cited in order to prevent the propagation of potentially misleading results and conclusions.

Is the work clearly and accurately presented and does it cite the current literature?

Partly

If applicable, is the statistical analysis and its interpretation appropriate?

No

Are all the source data underlying the results available to ensure full reproducibility?

Yes

Is the study design appropriate and is the work technically sound?

Partly

Are the conclusions drawn adequately supported by the results?

No

Are sufficient details of methods and analysis provided to allow replication by others?

Partly

Reviewer Expertise:

Electromagnetic interactions with biological systems; non-invasive therapies; microtubules; biophysics; high energy physics; particle physics.

I confirm that I have read this submission and believe that I have an appropriate level of expertise to state that I do not consider it to be of an acceptable scientific standard, for reasons outlined above.

## References

[1] : Selection of appropriate statistical methods for data analysis. *Ann Card Anaesth* .2019;22(3) : 10.4103/aca.ACA_248_18 297-301 10.4103/aca.ACA_248_18 31274493 PMC6639881

# Response

**Published:** 2023-10-27

**Authors:** Firman Alamsyah

**Affiliations:** Ctech Labs Edwar Technology, Indonesia

Dear Reviewer,

 We had difficulty carrying out statistical tests with 2 factors using the suggested test. We have tried it but it did not work. We had difficulty finding examples of the use of the test in published articles. We have also asked biostatisticians for help, but none of them can do it, including one of our colleagues who is the head of the Biostatistics Department at a university.

 Therefore, we carried out repeated statistical tests using only 1 factor, namely exposure to electric fields, by comparing the level of damage to kidney and liver tissues. We got different results from previous statistical tests. Based on the statistical results, we have replaced the graphs in Figures 3 and 5, and revised the Abstract, Results, and Discussion sections.

# Reviewer report: Reviewer response for version 1

**DOI:** 10.5256/f1000research.121655.r162014

**Published:** 2023-03-02

**Authors:** Michael Staelens

**Affiliations:** 1 Instituto de Física Corpuscular (IFIC), Paterna, Valencia, Spain

The manuscript, “Kidney and liver histology in tumour-induced rats exposed to non-contact electric fields”, by Alamsyah *et al*., reports new results on the effects of 100 kHz low-intensity non-contact electric fields on the histological profiles of kidney and liver samples taken from Sprague Dawley rats exposed to such fields (with and without tumour induction). Alternating electric fields with these particular parameters (low intensity and intermediate frequency) have become topical in oncology, especially over the last decade, due to the earlier discovery that these fields (also known as TTFields) greatly hinder the division of cancer cells. Several successful clinical trials on treating different types of cancer with TTFields have led to the subsequent FDA-approval of TTFields therapy for various cancers, such as glioblastoma. Consequently, many *in vivo* and *in vitro* studies on the efficacy of alternating electric field therapy have been conducted; however, safety studies are lacking in number and should grow in tandem with the efficacy studies. Thus, the study reported in the manuscript by Alamsyah *et al.* is both interesting and valuable to the scientific community (and a cancer research audience in particular); however, substantial major issues with the manuscript make the conclusions and, ultimately, the value and impact of the study uncertain. While I do believe the study has potential, the authors must revise the manuscript substantially before it should be indexed. Particularly, the literature review with regards to the Introduction and Discussion sections needs to be revisited and refocused; the statistical analysis used to determine the significance of the results does not apply, and a new analysis is required; and the very limited and overly general conclusions that do not appear to follow from the results and discussion presented in the manuscript must be stated more clearly to accurately reflect the results of the study. A complete list of my comments and concerns that must be addressed, both general and specific, are provided below.

**General Comments:**

1. There are many problems with the writing regarding grammar, punctuation, missing spaces, article usage problems, misspelled words, incorrect verb forms, faulty tense sequences, incomplete sentences, and several other sentences that simply do not make sense. In aggregate, these errors severely hinder the readability and clarity of the manuscript. I strongly recommend that the authors consider sending the manuscript to a professional English editing service, or at the very least, use one of the numerous free grammar and writing tools available online to assist with correcting many of the writing issues.
2. The manuscript mixes both U.S. and U.K. spellings, e.g., “ *tumor*” and “ *tumour*” are both used, as are “ *acclimatization*” and “ *randomisation*” (z vs. s). Please maintain one choice consistently in your writing.
3. The manuscript lacks coherence and does not communicate a cohesive story. Here are some examples of inconsistencies that negatively affect the cohesion:
  - In the “Results” part of the Abstract, it reads, “ *The damages in the kidney and liver caused by the **exposure **were **not significant**.* *”,* but then in the Discussion section, the following is stated “ *Since the **significant damages of the glomerulus were observed in the kidneys** of the **placebo (NIT)**…*”, followed by, “ *both DMBA administration and **exposure to non-contact electric fields affected the thickening of Bowman’s capsule***”, which certainly sounds like there were significant damages observed in the kidney samples taken from the group that only received exposure to the electric fields (NIT).
  - The need for studying the effects of electric fields-based cancer therapies on healthy tissues was motivated in the Introduction by pointing out that there might be interactions between the kidney/liver and **electrostatic **waves; however, the electric fields-based cancer therapies referenced and discussed in the manuscript (and that are actually used in a clinical setting) do not employ static fields, but rather alternating electric fields.
  - Several of the references cited in the Discussion section that are discussed with regards to the interpretation of the results studied **vastly different** parameters for the electric fields than those employed in the experiments reported in the manuscript and that are typically used to treat cancers with alternating electric field therapy (e.g., 575 V/cm in one of the studies cited vs. the low-intensity of only a few V/cm maximum for alternating electric field therapy).
4. The manuscript often mentions the **significance** of results/effects; however, none of these statements include the supporting quantitative results that suggest the differences obtained are, in fact, significant (or not). Consequently, the level of significance associated with these statements is also not clear to the reader (without reading through the data files provided alongside the manuscript). Please revise **all **these statements to include the quantitative results of the statistical analyses that support the stated significances. For example, the following sentence on page 5, “ *The main damage found in the kidney glomerular was the thickening of the Bowman capsule whose scores were **significant** in all treatment groups (1.12* *±* *0.56 for NIT, 1.16* *±* *0.74 for INT, and 1.24* *±* *0.59 for IT groups) compared to the control (NINT) group (0.88* *±* *0.56).* *”,* should be revised to include the corresponding *H*-values (or *p*-values, etc.) that supports the statement that these differences between scores were significant (“ *in all treatment groups compared to the control group*”). This makes it both clear to the reader that the differences between groups are indeed significant based on the statistical analysis results obtained and provides the reader with the quantitative results, so they also get a sense of **how significant** (or insignificant) each particular result is without having to dig through spreadsheets of data while reading the article.
5. The quality of the plots provided in Figures 2 and 4 is quite low. Additionally, the dots used to represent the data are very small and difficult to distinguish (when reading the article on a tablet at 100% magnification). Please consider reproducing these plots at a higher quality (600 DPI or greater) and with the points used to represent the data enlarged and spread out more clearly, so the individual points are distinguishable without needing to zoom in (which also leads to substantial blurring due to the low quality of the plots).

**Specific Comments:**

**Major concerns:**

1. The data analysis section states that the scoring results were analyzed statistically using the Kruskal–Wallis test; however, the scoring results were obtained for four different groups that **differed independently in two ways **(i.e., there were **two independent variables** involved—tumour induction (or not) and treatment (or not)). Consequently, the Kruskal–Wallis test, which is only applicable in the one-factor case, cannot be used. Due to this problem, the actual statistical significance of the obtained differences between the various groups studied is not clear, and the soundness of the conclusions drawn is uncertain. If you do require a non-parametric rank-based method, then consider the Scheirer–Ray–Hare extension of the Kruskal–Wallis test, which can be used in the case where a measure may be affected by two or more factors. Alternately, the (two-factor) aligned rank transform analysis of variance (ART-ANOVA) may also be suitable.
2. Moreover, the conclusions do not appear to be clearly supported by the results. This concern is not only due to the previous major comment noted but also due to the following:
  - The first conclusion drawn is that “ *The non-contact electric fields were **not harmful** to the renal and liver structure of tumour-induced rats"; *however, one of the outcomes described in the Results and Discussion sections was that the non-contact low-intensity electric fields significantly " *affected the thickening of Bowman's capsule*" (NINT versus NIT), which is apparently indicative of renal damage. Consequently, I am not sure that this general conclusion is completely supported by the results of this study.
  - The second conclusion drawn is that non-contact electric fields “ *may optimise/increase the renal function in normal rats*”. What results obtained in this study demonstrate an optimization and/or increase in renal function in normal rats exposed to low-intensity non-contact electric fields? The only discussion in the manuscript regarding renal function appears to be that provided on page 8; however, the discussion there mentions renal function impairment as a possible result of DMBA-induced inflammation and does not discuss electric fields. At the end of this paragraph, non-contact electric fields are mentioned: “ *non-contact electric fields decreased the number of inflammations and haemorrhages in the placebo (NIT) group, as shown in Figure 2*”; however, the statistical significance of this difference is not stated. Is the decrease being mentioned here actually meaningful statistically? And if so, how statistically significant was this decrease, and how exactly does this marginal decrease in observed inflammations and haemorrhages connect to the conclusion that renal function has been **optimized **due to treatment with non-contact electric fields? As far as I can tell, no tests or metrics that could be used to accurately evaluate any changes in kidney and/or liver function and that would support this particular conclusion were included in the experiments and analyses reported in the manuscript.
3. Why were damages also exhibited in the control (NINT) group? Naively, I would not have expected this outcome, and it is not explained or discussed in the manuscript.

Due to these major concerns, I do not believe that the conclusions stated in the manuscript are clearly supported by the results.

**Minor concerns:**

1. More details regarding both the electric field used in the experiments and how it was applied should be provided for clarity and reproducibility of the study:
  - How were the electric fields generated? What device was used (make, model, etc.)?
  - Were the electric fields static or alternating?
  - What was the duty cycle?
  - Given that the rats were exposed to non-contact electric fields, I assume that the electrodes were not fixed to the skin of the rats and that they received whole-body exposure to the electric fields. Is this true? Additionally, how many electrodes were used? where were they placed/attached? and with what orientation? In other words, how many electric fields were there, were they uniform or non-uniform, and with what directionality(ies)? 
  Please provide this information in the manuscript.
2. While the exposures were being performed, were there any other sources of electromagnetic radiation in the room that could be considered a source of interference?
3. On page 3, the last sentence in the Introduction states, “ *According to our knowledge, this is the first study investigating the abnormalities in the kidney and liver under exposure to 100 kHz intermediate frequency and low-intensity non-contact electric fields*”, which does appear to technically be true; however, a very similar ^1^ study was recently published that reports the results of analyzing histological profiles of vital organs of Sprague Dawley rats exposed to 150 kHz low-intensity non-contact electromagnetic radiation. It would be valuable and interesting to revise the discussion in the manuscript to compare and contrast your results and conclusions obtained versus those reported in this very similar study in the literature.
4. On page 3, a value of 18 V _pp_ is noted after stating that the electric fields used in the study are **low intensity**. The electric field intensity has units of electric potential per distance (V/m in SI units), not simply volts, which is the unit of electric potential. Please revise accordingly (including at the bottom of page 3, where the same issue occurs again).
5. On page 3, it is stated that “ *Although **non-contact electric fields-based therapy has the potential to treat cancer**, the safety of this kind of therapy when treating healthy tissues should be investigated. This is because injuries may occur after exposure to electric fields due to the dielectric property of the kidney and liver, which may interact with **electrostatic** **waves**. Therefore, it is important to investigate the abnormalities in the kidney and liver under exposure to **electric fields during cancer treatment*** *.*” The second sentence quoted here does not connect to the previous or subsequent sentences; the electric fields used in cancer treatment that this article alludes to, so-called TTFields, are **alternating** electric fields ( **time-varying**), whereas ***electrostatic *always refers to time-invariant electric fields**. Please revise.
6. Additionally, on page 3, the sentence “ *the proliferation of cancer cells was successfully inhibited under exposure to intermediate frequency and low-intensity electric fields*” should be revised to read “low-intensity alternating electric fields”. This statement would also benefit from more specificity, i.e., what types of cancer cells was this inhibition demonstrated for in the studies cited here and with what parameters for the electric fields employed (frequency, intensity, duration of exposure)?
7. Should the *y*-axis label for Figure 2d read “Congestion score” instead? (To maintain consistency with the other subfigures presented in Figure 2 and with Figure 4c.)
8. In Figure 4, the values of mean ± SD noted in each of the subfigures use inconsistent numbers of significant digits. Specifically, the values of the uncertainties are reported with greater precision than the mean values themselves, which indicates that the errors are known more precisely than the values and is very unusual.
9. Figures 2 and 4 include a footnote partially describing the meaning of the labels “a”, “b”, etc. as indicating significance; however, the difference between each of these labels is not clear. The exact meaning of “a”, “b”, etc. should be clarified in the notes provided under Figures 2 and 4. Additionally, while it is clear that, for example, in Figure 2a, the NINT group has the label “a” to denote that it is significant, it is not clear what it is being compared to, i.e., significant compared to which group(s)? Lastly, please change “ *different words a, b, c*” to read “different letters” or “different labels”.
10. In the second paragraph of the Discussion on page 7, it is mentioned that “ *some biological effects of exposure to electric fields (0.6 and 340 kV/m) were revealed in humans and vertebrates, but no histological abnormalities were found in the organs, including the kidneys.*” Comparing with this study does not make much sense for the following reasons:
  - The particular study cited here evaluated the biological effects of **static electric fields**, which are not the same as the **alternating** electric fields exploited in cancer therapy, and that the manuscript purportedly studies the effects of.
  - The parameters stated (0.6 and 340 kV/m) corresponding to this particular study that is being referenced are **NOT** consistent with the **low-intensity **electric fields used in cancer therapy (approx. 1–3 V/cm) and, again, that the manuscript appears to be studying.
11. The abbreviation ECCT is not defined anywhere in the manuscript.
12. The first sentence in the last paragraph of the Discussion section (page 9) does not make sense. Specifically, what does “ *Based on the evidence for the efficacy and safety of normal tissues and organs...*” mean? I believe that rather than describing evidence on the “efficacy and safety of a normal tissue”, what is trying to be said here is something like, “Based on the evidence for the efficacy and safety of non-contact low-intensity electric fields regarding normal tissues and organs...”, or something along these lines. Please rephrase this sentence accordingly.
13. The statement regarding the role of the funders, “ *The funders had no role in study design, data collection and analysis, decision to publish, or preparation of the manuscript.*”, should also mention the role of the funders regarding the interpretation of the data/results.

Is the work clearly and accurately presented and does it cite the current literature?

Partly

If applicable, is the statistical analysis and its interpretation appropriate?

No

Are all the source data underlying the results available to ensure full reproducibility?

Yes

Is the study design appropriate and is the work technically sound?

Partly

Are the conclusions drawn adequately supported by the results?

No

Are sufficient details of methods and analysis provided to allow replication by others?

Partly

Reviewer Expertise:

Electromagnetic interactions with biological systems; microtubules; biophysics; high energy physics; particle physics.

I confirm that I have read this submission and believe that I have an appropriate level of expertise to state that I do not consider it to be of an acceptable scientific standard, for reasons outlined above.

## References

[1] : Effects of Intermediate Frequency (150 kHz) Electromagnetic Radiation on the Vital Organs of Female Sprague Dawley Rats. *Biology (Basel)* .2023;12(2) : 10.3390/biology12020310 10.3390/biology12020310 36829585 PMC9952889

# Response

**Published:** 2023-03-23

**Authors:** Firman Alamsyah

**Affiliations:** Ctech Labs Edwar Technology, Indonesia

We thank you for all the comments and suggestions and we will revise our paper. Below are our answers to the comments and suggestions provided in order.

 General comments

 1. We have used a professional English editing service (PaperTrue) before submitting this article. We will do one more proofreading.

 2. We will use UK spelling in our revised article.

 3. We will revise the abstract so that it is coherent with the contents of the paper. We will replace some of the references in the Discussion section. Previously, we did not find any references in accordance with the study conducted.

 4. In the Data Analysis section and in the Figures, we have mentioned a p<0.05 value for a significant difference among groups. We will rewrite it for each data in the Results section.

 5. We will fix Figures 2 and 4.

 Specific comments

 Major concerns

 1. We will evaluate the statistical tests performed.

 2. We will revise the Conclusions section to suit the Results and Discussion sections.

 3. Damage to the kidneys and liver of the rats in the control group (NINT) cannot be predicted because we have excluded rats with symptoms of illness as stated in the article. Rats were also randomly selected for each group as stated also in article.

 For damage to the kidney in the NINT group, if we look at the interstitial tissue, a score below 2 indicates that there is little inflammation or hemorrhage. Inflammation is part of the activation of the immune system in response to acute or chronic kidney injury which can be caused by pathogens that enter the rat’s body (Imig & Ryan, 2013).

 For damage to the liver in the NINT group, if we look at the hemorrhagic and congestion scores which are below 1, this indicates that there is little or no damage to the liver. For a cellular damage score below 2, this indicates reversible damage with less than 15% necrosis. Liver hepatocytes have many vital functions, so they can proliferate extensively, which allows efficient regeneration of the liver for reversible damage (Chen et al., 2020). In addition, the liver itself is a very vulnerable organ due to its size and is the organ most frequently injured after abdominal trauma (Bilgic et al., 2014).

 Minor concerns

 1. We will provide more detailed information about the electric fields used in the experiment in our revised article.

 2. The experiment was carried out in a special room which only contained experimental animal cages.

 3. We will revise the Discussion section using the appropriate references.

 4. We will include electric fields intensity data in our revised article.

 5. We will revise this section (page 3).

 6. We will revise this section (page 3).

 7. We will revise Figure 2d.

 8. We will revise Figure 4.

 9. We will revise Figures 2 and 4.

 10. We will revise the Discussion section using the appropriate references.

 11. We will write what ECCT stands for in our revised article.

 12. We will rephrase this sentence. There may be phrases lost in the editing process.

 13. We will add the statement regarding the role of the funders in the interpretation of the data/results. 

 References

 Bilgiç I, Gelecek S, Akgün AE, *et al*.: Evaluation of liver injury in a tertiary hospital: a retrospective study. *Ulus Travma Acil Cerrahi Derg*. 2014; 20(5): 359-365.

 Chen F, Jimenez RJ, Sharma K, *et al*.: Broad Distribution of Hepatocyte Proliferation in Liver Homeostasis and Regeneration. *Cell Stem Cell*. 2020; 26(1):27-33

 Imig JD, Ryan MJ: Immune and Inflammatory Role in Renal Disease. *Compr. Physiol*. 2013; 3(2): 957-976.

# Reviewer report: Reviewer response for version 1

**DOI:** 10.5256/f1000research.121655.r162015

**Published:** 2023-02-27

**Authors:** Chandran Nadarajan

**Affiliations:** 1 School of Medical Sciences, Hospital Universiti Sains Malaysia, Kota Bharu, Malaysia

**Editorial Note from F1000Research – 06/03/2023:**

*This report has been updated after the reviewer informed the editorial team that they had further comments after their initial review was published. The changes are due to some factors which were not assessed in the initial review and were picked up when the reviewer read the article again. This update has not changed the ‘Approved’ status that the reviewer originally assigned.*

 This study touches on the effect of a new technology being introduced in oncology and answers some of the questions regarding the safety issue of the device. This information is critical to further utilize this device in the general public. 

 Some additional information from the authors could elevate the write-up:

1. Why do the authors choose the voltage 100kHz and 18v, specifically? Were the preliminary results referred to published? Are there any other articles supporting the usage of this voltage and frequency? 
2. Is there any statistical analysis done? This will help strengthen the conclusion. 
3. Why was there damage in the kidney interstitial tissue and liver damage in the NINT group?

In addition, some of the articles referred to use different voltage and frequency levels; therefore, it couldn't be a direct comparison of this study. Such correlation needed to be taken with caution.

 Overall, this study tries to answer the safety aspect of this non-contact electrical field therapy and highlights some promising changes.

 --------------------------------------------------------------------------

 First of all, I maintain that the paper is quite impressive and constructive with a novel idea. However, I would suggest a few additional points:

1. Figures 1 and 3 are very small, with many icons. Please enhance them.
2. Figures 2, and 4 suggest changing to the Whisker box plot and being made bigger. It will be better.
3. Utilization of rank order, like Mann Whitney, might help to bring forward the idea better.
4. Conclusion is a little too short and needs to be improved. Adding limitations and further direction might help. This can be done in the discussion.
5. IRB date, number, and place should be shown.
6. In several places, language is unclear, ambiguous, or confusing. It is necessary to use a professional editing service to improve it before indexing.
7. I think the title "Effects of non-contact electric fields on kidney and liver histology in tumour-induced rats." Might be more appropriate.

Is the work clearly and accurately presented and does it cite the current literature?

Yes

If applicable, is the statistical analysis and its interpretation appropriate?

Partly

Are all the source data underlying the results available to ensure full reproducibility?

Yes

Is the study design appropriate and is the work technically sound?

Yes

Are the conclusions drawn adequately supported by the results?

Partly

Are sufficient details of methods and analysis provided to allow replication by others?

Yes

Reviewer Expertise:

Interventional oncology, interventional and diagnostic radiology

I confirm that I have read this submission and believe that I have an appropriate level of expertise to confirm that it is of an acceptable scientific standard.

# Response

**Published:** 2023-03-01

**Authors:** Firman Alamsyah

**Affiliations:** Ctech Labs Edwar Technology, Indonesia

I will answer the reviewer's comments sequentially in the order of the comments.

 1. We have conducted an *in vitro* study using various frequencies and intensities, but this study has not been published. We used 100 kHz and 18 Vpp for *in vivo* study because this frequency and intensity gave the best results in *in vitro* study, where 28-39% of breast cancer cells died (Alamsyah et al., 2015). In the preliminary *in vivo* study using 9 mice, the frequency of 100 kHz and intensity of 18 Vpp also gave good results, where the tumor size was reduced by more than 67% and the results of histopathological analysis on normal skin and breast tissue, showed no damage (Alamsyah *et al.*, 2015).

 2.  Yes, we did the statistical analysis and it has been written in the article in the Data Analysis section. Kidney and liver scoring data, as well as statistical analysis can be seen in the links provided in the Data Availability section of the article.

 3. Damage to the kidneys and liver of the rats in the control group (NINT) cannot be predicted, because we have excluded rats with symptoms of illness as stated in the article. Rats were also randomly selected for each group as stated also in article.

 For damage in the renal interstitial tissue of the NINT group, a score below 2 indicated that there was little inflammation or hemorrhage. Inflammation is part of the activation of the immune system in response to acute or chronic kidney injury which can be caused by pathogens that enter the rat’s body (Imig & Ryan, 2013).

 For damage in the liver of the NINT group, if we look at the hemorrhagic and congestion scores which are below 1, this indicated that there was little or no damage to the liver. For a cellular damage score below 2, this indicated reversible damage with less than 15% necrosis. Liver hepatocytes have many vital functions, so they can proliferate extensively, which allows efficient regeneration of the liver for reversible damage (Chen *et al.*, 2020). In addition, the liver itself is a very vulnerable organ due to its size and is the organ most frequently injured after abdominal trauma (Bilgic *et al*., 2014). Rats are active animals and may chase or fight each other in communal cages which can cause trauma to their body (Steimer, 2011).

 4. We found no reference to kidney or liver damage at intermediate frequency and low intensity electric fields, especially 100 kHz and 18 Vpp. We have also stated this in the article. In the Introduction section, we have also stated that this is the first study to investigate abnormalities in the kidney and liver under exposure to a intermediate frequency of 100 kHz and a low intensity non-contact electric field.

 We will add necessary information to our article from the answers to the reviewer's questions.

 Thank you.

 References

 Alamsyah F, Ajrina IN, Dewi FN, et al.: Antiproliferative Effect of Electric Fields on Breast Tumor Cells In Vitro and In Vivo. Indones. J. Cancer Chemoprev. 2015; 6(3): 71–77.

 Bilgiç I, Gelecek S, Akgün AE, *et al*.: Evaluation of liver injury in a tertiary hospital: a retrospective study. *Ulus Travma Acil Cerrahi Derg*. 2014; 20(5): 359-365.

 Chen F, Jimenez RJ, Sharma K, *et al*.: Broad Distribution of Hepatocyte Proliferation in Liver Homeostasis and Regeneration. *Cell Stem Cell*. 2020; 26(1):27-33

 Imig JD, Ryan MJ: Immune and Inflammatory Role in Renal Disease. *Compr. Physiol*. 2013; 3(2): 957-976.

 Steimer T **: **Animal models of anxiety disorders in rats and mice: some conceptual issues. *Trans Res*. 2011; 495-506.

# Response

**Published:** 2023-03-20

**Authors:** Firman Alamsyah

**Affiliations:** Ctech Labs Edwar Technology, Indonesia

We thank you for the additional suggestions and we will revise our paper. Below are our answers to the suggestions provided in order.

 1. We will enhance Figure 1 and Figure 3 in our revision.

 2. We will consider to use the Whisker box plot in Figure 2 and Figure 4.

 3. We have conducted Mann-Whitney test (α=0.05) in our statistical analysis.

 4. We will improve the conclusions of our study and we will discuss the limitations of this study. We have written further directions of this study at the end of the discussion section.

 5. IRB date, number, and place have been written in the ethical approval section.

 6. We have used a professional editing service (PaperTrue) before submitting this article. We will do one more proofreading.

 7. We will improve the title of this article.
